# Supplementary material for: 3D genome mapping identifies subgroup-specific chromosome conformations and tumor-dependency genes in ependymoma
Source: Nat Commun. 2023 Apr 21;14:2300. doi: 10.1038/s41467-023-38044-0 (PMC10121654; doi:10.1038/s41467-023-38044-0)
Supplement: Supplementary file 1 — Supplementary Information [file 41467_2023_38044_MOESM1_ESM.pdf]

Supplementary Figures

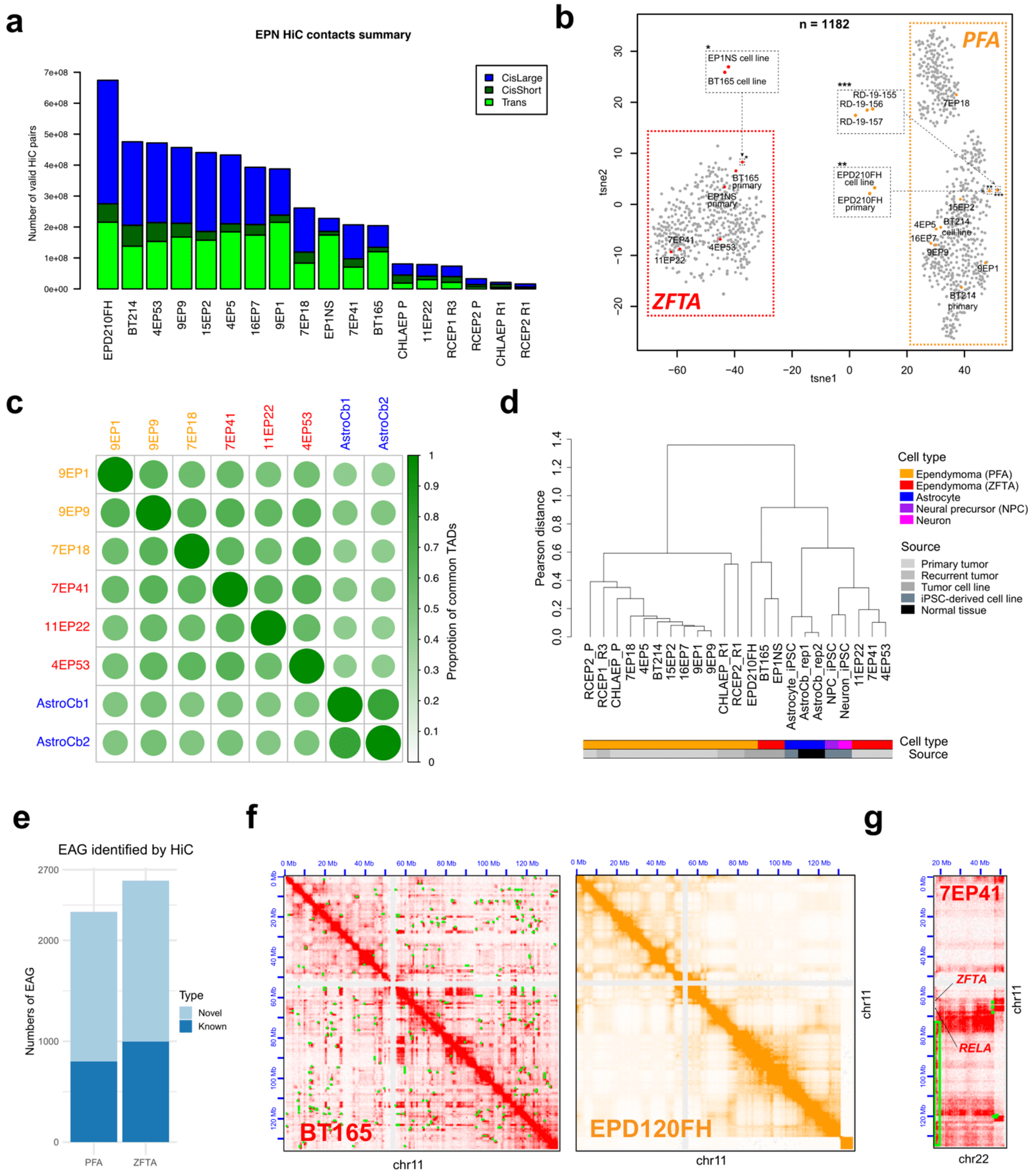

Supplementary Figure 1

a) The number of DNA contacts obtained in the individual ependymoma samples for the Hi-C data.

b) The tSNE dimensionality reduction visualization of DNA methylation data from a cohort of ependymoma samples (n=1,182) separates PFA and ZFTA ependymomas into separate groups. The samples analyzed in this study by Hi-C are highlighted in red and orange. Blocks marked with an asterisk show a magnification of selected samples, for better visualization.

c) Proportions of topologically associated domains (TADs) shared between ependymoma tumors. The comparison is performed among PFA (n=3) and ZFTA (n=3) ependymoma tumors that were also included in previous enhancer-mapping study. Hi-C data from two normal cerebellum astrocytes provided in the ENCODE database were included as controls. The mean proportion of common TADs across all samples is 0.569, which is smaller in comparison to the mean proportions of common TADs among samples of the same group (PFA: 0.627, ZFTA: 0.63 and cerebellar astrocytes: 0.784).

d) Unsupervised clustering of Hi-C data, including all tumor samples and cell lines as well as unrelated Hi-C data from different cell types of the neural lineage such as cerebellar astrocytes, neural precursor cells (NPC) and astrocytes derived from induced pluripotent stem cells (PSCs). The results demonstrate that the Hi-C data quality of all tumor samples allows for a clear separation by ependymoma subgroup. While the separately processed cell lines show greater differences from the tumor samples, they also cluster by subgroup, i.e. the two ZFTA ependymoma lines EP1NS and BT165 show high similarities to each other.

e) The integrative analysis of Hi-C, enhancer and gene expression data reveals that substantially more genes as previously reported18 are regulated by proximal and distal ependymoma enhancers (the proportion of novel vs. previously reported enhancer regulated genes is ~ 1.85 for PFA and ~ 1.6 for ST-RELA).

f) Hi-C map of chromosome 11 shattered by chromothripsis in a patient-derived ZFTA sample (ZFTA BT165, left) compared to a stable chromosome 11 in a PFA sample (EPD210FH, right). Green boxes indicate structural variants detected by analyzing SVs based on the Hi-C data.

g) Structural variants in RELA tumors are not limited to chromosome 11 but also involve other chromosomes. Shown is an inter-chromosomal structural variant that includes chr11 and chr22 in the ZFTA ependymoma sample 7EP41.

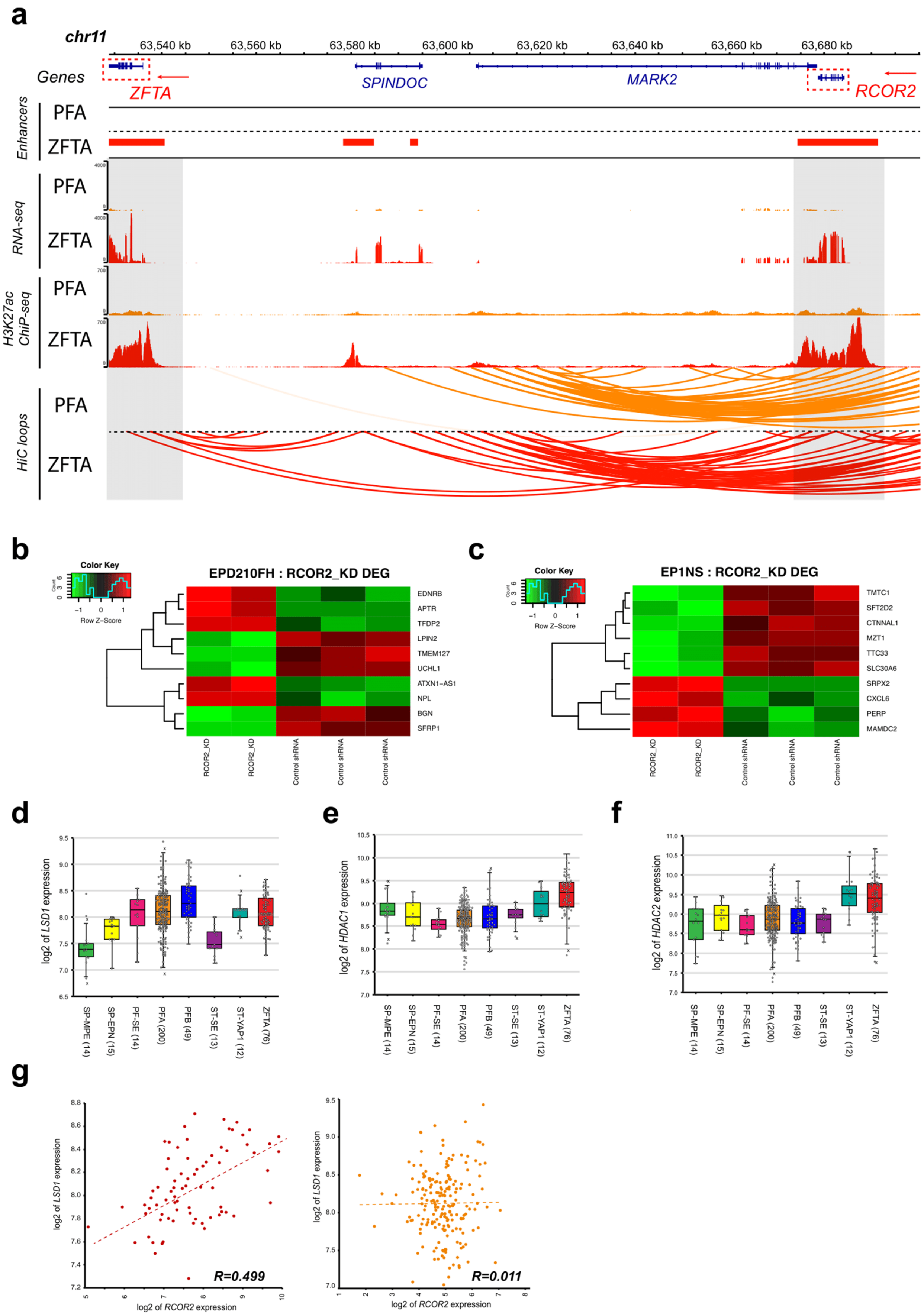
a
chr11
63,540 kb
63,560 kb
63,580 kb
63,600 kb
63,620 kb
63,640 kb
63,660 kb
63,680 kb
Genes
ZFTA
SPINDOC
MARK2
RCOR2
Enhancers
PFA
ZFTA
RNA-seq
PFA
ZFTA
H3K27ac ChIP-seq
PFA
ZFTA
HiC loops
PFA
ZFTA
b
Color Key
Row Z-Score
EPD210FH : RCOR2_KD DEG
EDNRB
APTR
TFDP2
LPIN2
TMEM127
UCHL1
ATXN1-AS1
NPL
BGN
SFRP1
RCOR2_KD
RCOR2_KD
Control shRNA
Control shRNA
Control shRNA
c
Color Key
Row Z-Score
EP1NS : RCOR2_KD DEG
TMTC1
SFT2D2
CTNNAL1
MZT1
TTC33
SLC30A6
SRPX2
CXCL6
PERP
MAMDC2
RCOR2_KD
RCOR2_KD
Control shRNA
Control shRNA
Control shRNA
d
log2 of LSD1 expression
e
log2 of HDAC1 expression
f
log2 of HDAC2 expression
SP-MPE (14)
SP-EPN (15)
PF-SE (14)
PFA (200)
PFB (49)
ST-SE (13)
ST-YAP1 (12)
ZFTA (76)
g
log2 of LSD1 expression
log2 of RCOR2 expression
R=0.499
log2 of LSD1 expression
log2 of RCOR2 expression
R=0.011

Supplementary Figure 2

a) Genome browser visualization including the ZFTA and RCOR2 genes shows enhancers that interact with RCOR2 by ZFTA ependymoma-specific DNA loops. Tracks for RNA-seq, H3K27ac and Hi-C derived DNA loops were obtained by merging data from PFA (9EP1,9EP9, 7EP18) or ZFTA (11EP22, 4EP53, 7EP41) tumors, respectively.

b) Heatmap of the top 10 differentially expressed genes (limma p-val <0.001) observed upon RCOR2 knock down compared to control experiments in the PFA cell line EPD210FH.

c) Heatmap of the top 10 differentially expressed genes (limma p-val <0.001) observed upon RCOR2 knock down compared to control experiments in the ZFTA cell line EP1NS.

d-f) Boxplots of *LSD1*, *HDAC1* and *HDAC2* gene expression across ependymoma subgroups using Affymetrix gene expression data (n=393, ZFTA vs all other tumor classes limma p-values: 0.85, 1.48-13, 4.9e-16). The center line, box limits, whiskers, and points indicate the median, upper/lower quartiles, 1.5× interquartile range and outliers, respectively.

g) Correlation between RCOR2 and LSD1 in ZFTA (left side, n=76, cor=0.499, p-val= 4.52e-06) and PFA ependymoma samples (right side, n=200, cor=0.011, p-val= 0.880).

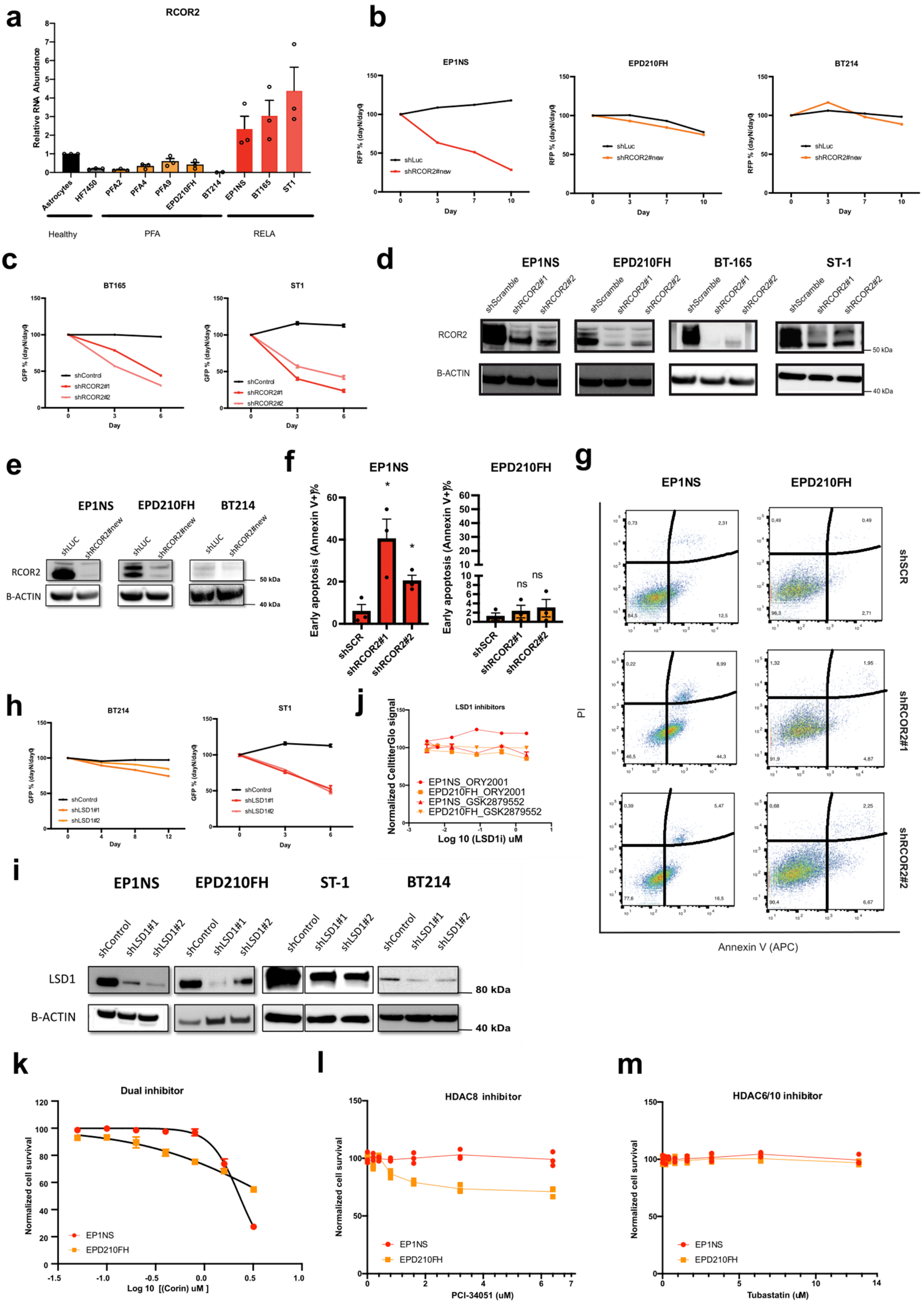
a
RCOR2
Relative RNA Abundance
Astrocytes
HF7450
PFA2
PFA4
PFA9
EPD210FH
BT214
EP1NS
BT165
ST1
Healthy
PFA
RELA
b
EP1NS
EPD210FH
BT214
RFP % (dayN/day0)
shLuc
shRCOR2#new
Day
c
BT165
ST1
GFP % (dayN/day0)
shControl
shRCOR2#1
shRCOR2#2
Day
d
EP1NS
EPD210FH
BT-165
ST-1
shScramble
shRCOR2#1
shRCOR2#2
RCOR2
B-ACTIN
50 kDa
40 kDa
e
EP1NS
EPD210FH
BT214
shLUC
shRCOR2#new
RCOR2
B-ACTIN
50 kDa
40 kDa
f
EP1NS
EPD210FH
Early apoptosis (Annexin V+)%
shSCR
shRCOR2#1
shRCOR2#2
ns
g
EP1NS
EPD210FH
shSCR
shRCOR2#1
shRCOR2#2
PI
Annexin V (APC)
h
BT214
ST1
GFP % (dayN/day0)
shControl
shLSD1#1
shLSD1#2
Day
j
LSD1 inhibitors
Normalized CelltiterGlo signal
EP1NS_ORY2001
EPD210FH_ORY2001
EP1NS_GSK2879552
EPD210FH_GSK2879552
Log 10 (LSD1i) uM
i
EP1NS
EPD210FH
ST-1
BT214
shControl
shLSD1#1
shLSD1#2
LSD1
B-ACTIN
80 kDa
40 kDa
k
Dual inhibitor
Normalized cell survival
EP1NS
EPD210FH
Log 10 [(Corin) uM]
l
HDAC8 inhibitor
Normalized cell survival
EP1NS
EPD210FH
PCI-34051 (uM)
m
HDAC6/10 inhibitor
Normalized cell survival
EP1NS
EPD210FH
Tubastatin (uM)

Supplementary Figure 3

a) RCOR2 transcription levels measured by qPCR in PFA, ZFTA and control cell lines used for functional validation experiments. Data are presented as mean ± SD from n=3 independent experiments per cell line.

b) Cell growth assays in ZFTA (EP1NS) and PFA cell lines (EPD210FH and BT214) with a third shRNA against RCOR2 or control shRNA (Luciferase). Cell growth are tracked by FACS as the percentage of shRNA expressing vectors tagged by RFP over indicated time. Results are normalized to day 0 and data represent mean from n=2 independent experiments per cell line.

c) Cell growth assays in additional ZFTA cell lines (BT165 and ST1) expressing shRNA against RCOR2 or control shRNA (Scramble). Cell growth are tracked by FACS as the percentage of shRNA expressing vectors tagged by GFP over indicated time. Results are normalized to day 0 and data represent mean from n=2 independent experiments for BT165 and mean ± SD from n=3 independent experiments for ST1.

d) Knock-down of RCOR2 expression in ZFTA (EP1NS, BT-165 and ST-1) and PFA (EPD210FH) cell lines. Representative western blots show protein levels 4 days post infection for each shRNA. B-actin is used as a loading control (n=3 for EP1NS, EPD210FH; n=2 BT-165; n=1for ST-1).

e) RCOR2 protein levels by Western blots after 4 days post infection with indicated shRNAs are measured. B-actin is used as a loading control.

f-g) RCOR2 depletion results in apoptotic cell death detected by AnnexinV-PI staining by FACS for n=3 independent experiments. Error bars represent mean ±SD and derived from unpaired two tailed t-test calculated by GraphPad (p=0,0271). Representative FACS plot is shown in panel g.

h) Cell growth of PFA (BT214) and ZFTA (ST1) cell lines expressing shRNA against LSD1 or control shRNA (Scramble). Cell growth are tracked by FACS as the percentage of shRNA expressing vectors tagged by GFP over indicated time. Results are normalized to day 0 and data represent mean from n=2 independent experiments for BT214 and mean ± SD from n=3 independent experiments for ST1.

i) Knock-down of LSD1 expression in ZFTA (EP1NS and ST-1) and PFA (EPD210FH and BT214) cell lines. Representative Western blots show protein levels 4 days post infection with indicated shRNAs. B-actin is used as a loading control (n=3 for EP1NS, EPD210FH; n=2 for BT-165; n=1 for ST-1).

j) Dose response curves of LSD1 inhibitors treatment with either ORY-2001 or GSK2879552 of ZFTA (EP1NS) and PFA (EPD210FH) ependymoma spheroids over a 96- hour time –course using Celltiter–Glo cell viability assay (n=3).

k) Dose response curves of single-compound treatment with Corin of ZFTA (EP1NS, BT165 and ST-1) and PFA (EPD210FH, BT214) ependymoma spheroids over a 72-hour time-course using Celltiter-Glo cell viability assays. For each sample the results are presented as percentage of the Luminescence signal from control condition (DMSO as a vehicle). Data are presented as SD from three independent experiments per cell line (n=3).

l) Dose response curves of the HDAC8 inhibitor PCI-34051 in ZFTA (EP1NS) and PFA (EPD210FH) ependymoma spheroids over a 72-hour time-course using Celltiter-Glo cell viability assays. For each sample, the results are presented as percentage of the Luminescence signal from control condition (DMSO as a vehicle). Data are presented as SD from three independent experiments per tumor type.

m) Dose response curves of the HDAC 6/10 inhibitor Tubastatin in ZFTA (EP1NS) and PFA (EPD210FH) ependymoma spheroids over a 72-hour time-course using Celltiter-Glo cell viability assays. For each sample, the results are presented as percentage of the Luminescence signal from control condition (DMSO as a vehicle). Data are presented as SD from three independent experiments per tumor type (n=3).

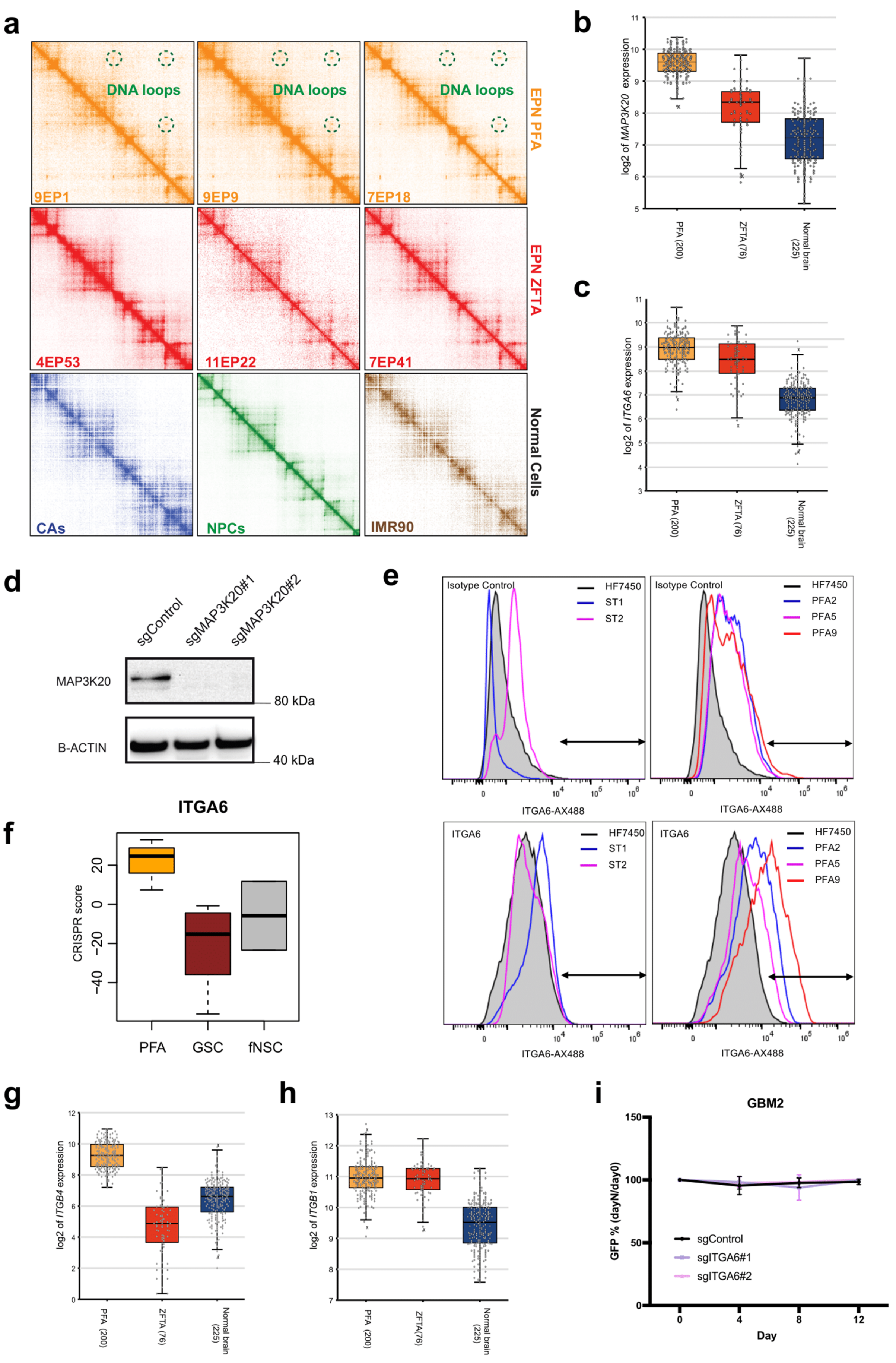
a
DNA loops
EPN PFA
9EP1
9EP9
7EP18
EPN ZFTA
4EP53
11EP22
7EP41
Normal Cells
CAs
NPCs
IMR90
b
log2 of MAP3K20 expression
PFA (200)
ZFTA (76)
Normal brain (225)
c
log2 of ITGA6 expression
d
sgControl
sgMAP3K20#1
sgMAP3K20#2
MAP3K20
80 kDa
B-ACTIN
40 kDa
e
Isotype Control
ITGA6
HF7450
ST1
ST2
PFA2
PFA5
PFA9
ITGA6-AX488
f
ITGA6
CRISPR score
PFA
GSC
fNSC
g
log2 of ITGB4 expression
h
log2 of ITGB1 expression
i
GBM2
GFP % (dayN/day0)
sgControl
sgITGA6#1
sgITGA6#2
Day

Supplementary Figure 4

a) DNA interaction matrices derived from Hi-C data wherein a ~5 million base pair segment of chromosome 2 (172,407,031-177,907,030 bp) is aligned along the diagonals. Off-diagonal signals indicate DNA interactions between different genomic sites. The chromatin complex at the *ITGA6* locus recurrently forms in all PFA EPN tumors analyzed (shown for samples 9EP1, 9EP9, and 7EP18 in the top row). This chromatin complex is not present in ZFTA ependymoma tumors (middle row). Moreover, normal human cell types analyzed by the ENCODE and PsychENCODE consortia, such as cerebellar astrocytes (CA), neural progenitor cells (NPCs) and embryonic fibroblasts (IMR90, bottom row), do not show signs of similar DNA interactions, suggesting that this chromatin complex is characteristic for PFA ependymoma tumors.

b-c) Differential gene expression analysis of Affymetrix array data identified MAP3K20 and ITGA6 as significantly (limma p-vals: 1.39e-22, 2.29e-06) upregulated in PFA compared to ZFTA ependymoma tumors and normal brain samples (n=200 PFA, n=76 ZFTA, and n=225 normal human brain samples). The box plot center line, box limits, whiskers, and points indicate the median, upper/lower quartiles, 1.5× interquartile range and outliers, respectively.

d) Western blots of lysates from the EPD210FH cell line infected with different guide RNAs. Cells were collected 5 days after infection. Data shows knock-out efficiency of guide RNAs targeting MAP3K20 on protein level compared to control guide RNA (n=1).

e) Histograms derived from FACS analysis of live ITGA6-Alexa-488 stained live cells from cultured ependymoma (ST1-2, ST-RELA cell lines, and PFA2, 5, 9, PFA cell lines) and normal human fetal neuronal stem cell line (HF7450).

f) *ITGA6* has been observed as an essential gene specifically in PFA ependymoma cell lines compared to glioblastoma stem cells (GSCs) and fetal neural stem cells (fNSCs) in a published CRISPR-Cas9 knock-out screen (Michealraj et al., 2020b). The box plot center line, box limits, whiskers, and points indicate the median, upper/lower quartiles, 1.5× interquartile range and outliers, respectively.

g-h) Expression of two alternative ITGA6 heterodimer partner proteins ITGB1 and ITGB4. While *ITGB1* and *ITGB4* are highly expressed in PFA and ZFTA ependymoma tumors compared to normal brain samples, only *ITGB4* is significantly upregulated (limma p-val: 4.03e-09) in PFA compared to ZFTA ependymoma tumors, suggesting that the integrin α6β4 heterodimer is the functional form relevant for PFA ependymoma tumors (n=200 PFA, n=76 RELA, and n=225 normal human brain samples). The box plots center line, box limits, whiskers, and points indicate the median, upper/lower quartiles, 1.5× interquartile range and outliers, respectively.

i) Genetic (CRISPR-Cas9) time-course knockout of *ITGA6* in glioblastoma (GBM2) cells using a control sgRNA and two individual sgRNA constructs. All constructs are GFP tagged and GFP positive cells are sorted by FACS. Results are normalized to day 0 and error bars represent mean±SD from n=3 independent experiments.

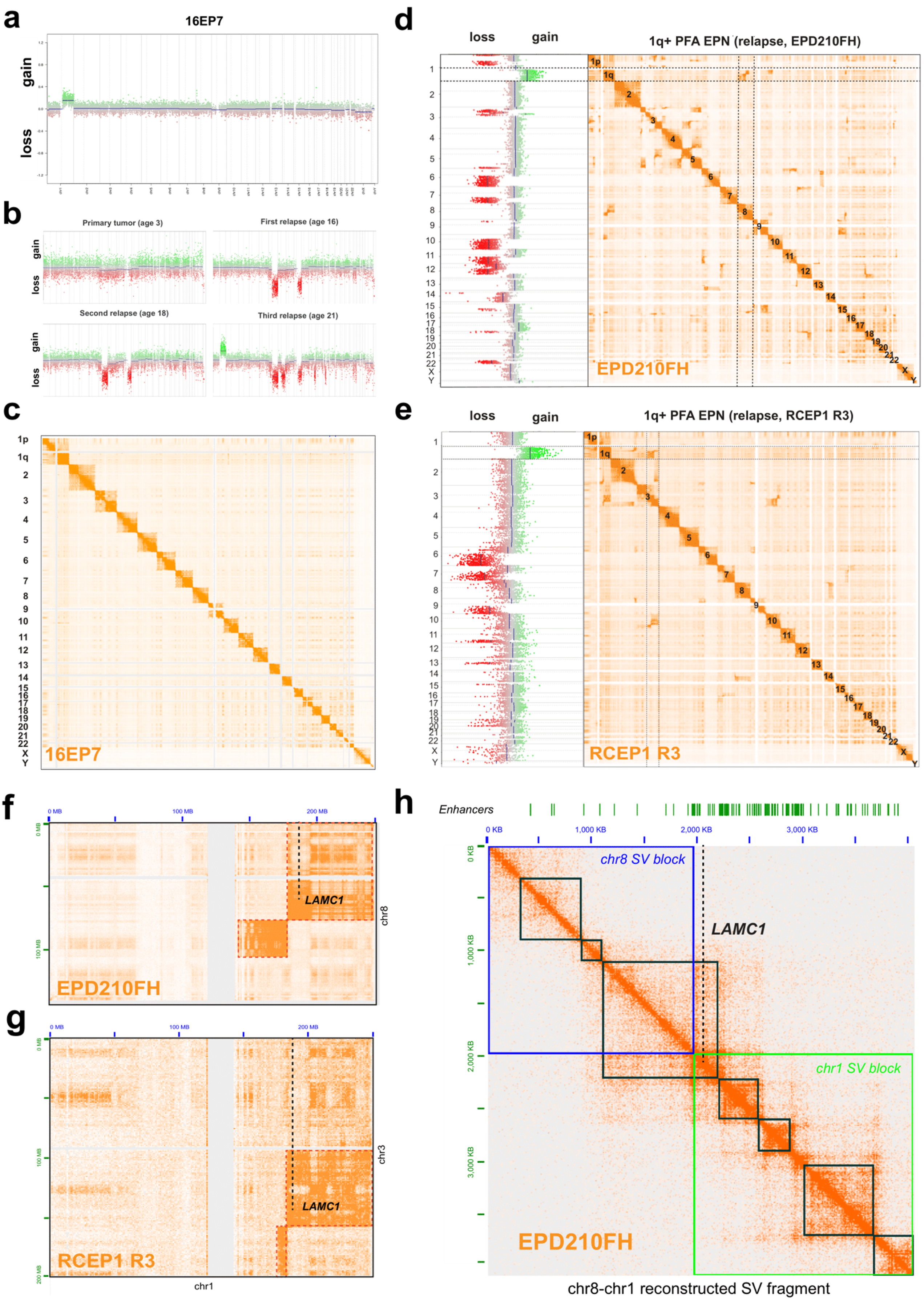
a
16EP7
gain
loss
b
Primary tumor (age 3)
First relapse (age 16)
Second relapse (age 18)
Third relapse (age 21)
c
16EP7
d
loss
gain
1q+ PFA EPN (relapse, EPD210FH)
EPD210FH
e
loss
gain
1q+ PFA EPN (relapse, RCEP1 R3)
RCEP1 R3
f
LAMC1
chr8
EPD210FH
g
LAMC1
chr3
RCEP1 R3
chr1
h
Enhancers
0 KB
1,000 KB
2,000 KB
3,000 KB
chr8 SV block
LAMC1
chr1 SV block
EPD210FH
chr8-chr1 reconstructed SV fragment

Supplementary Figure 5

a) Example of a copy number profile of a primary PFA ependymoma tumor (16EP7) harboring a 1q gain. The CNV profile was obtained from DNA methylation array data.

b) Copy number profiles of a primary and three recurrent PFA ependymoma tumors from the same patient (RCEP1 R3). The CNV profiles were obtained from DNA methylation array data.

c) Visualization of Hi-C data obtained from a primary PFA ependymoma tumor (16EP7) harboring a 1q gain shows frequent DNA interactions within chromosomes but no DNA interactions indicative of inter-chromosomal structural variants.

d) The conventional copy number profile of a PFA relapse sample (EPD210FH) shows high genomic instability including gain of chromosome arm 1q (presented vertically on the left). The genome-wide DNA interaction (Hi-C) map obtained from the same sample identifies complex inter-chromosomal structural variants including an inversion that involves chr1q and chr8. These trans-SVs reveal the complexity of genomic rearrangements underlying some copy-number gains and losses observed by common copy number variation analyses.

e) The conventional copy number profile of a PFA relapse sample (RCEP1 R3, third relapse) shows high genomic instability including gain of chromosome arm 1q (presented vertically on the left). The genome-wide DNA interaction map obtained from the same sample Hi-C data identifies complex inter-chromosomal structural variants including an inversion that involves chr1q and chr3.

f) Visualization of a translocation between chr1q and chr8 in the PFA ependymoma relapse sample EPD210FH.

g) Visualization of a translocation between chr1q and chr3 in the PFA ependymoma relapse sample RCEP1 R3.

h) Re-construction of the structural variant that involves chr1q and chr8 in the PFA relapse sample EPD210FH using Hi-C data in dual view. This structural variant results in the formation of a neo-TAD that places the LAMC1 gene locus in a new regulatory environment.

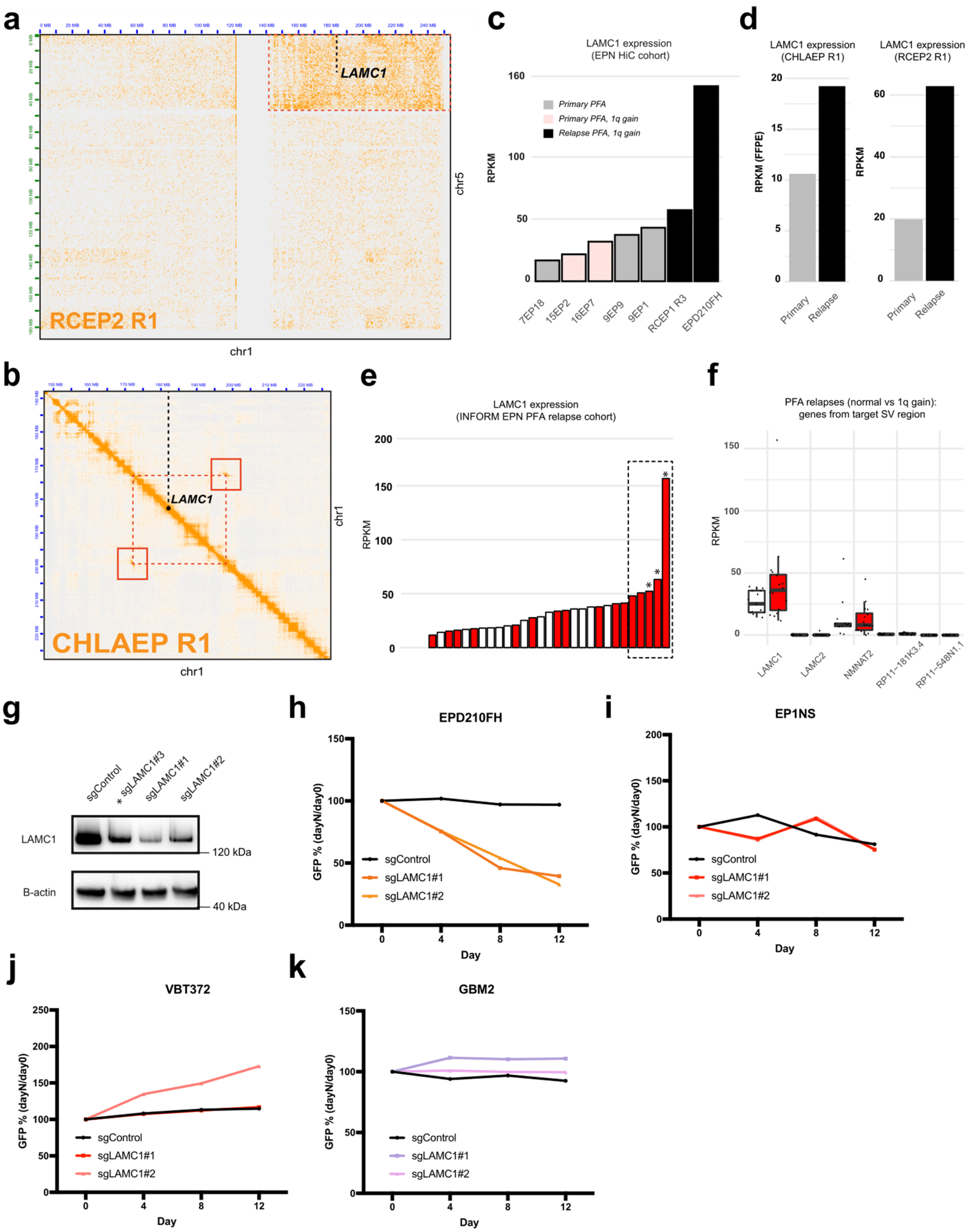

a
LAMC1
RCEP2 R1
chr1
chr5
c
LAMC1 expression
(EPN HiC cohort)
Primary PFA
Primary PFA, 1q gain
Relapse PFA, 1q gain
RPKM
7EP18
15EP2
16EP7
9EP9
9EP1
RCEP1 R3
EPD210FH
d
LAMC1 expression
(CHLAEP R1)
RPKM (FFPE)
Primary
Relapse
LAMC1 expression
(RCEP2 R1)
RPKM
b
LAMC1
CHLAEP R1
chr1
e
LAMC1 expression
(INFORM EPN PFA relapse cohort)
RPKM
f
PFA relapses (normal vs 1q gain):
genes from target SV region
RPKM
LAMC1
LAMC2
NMNAT2
RP11-181K3.4
RP11-548N1.1
g
sgControl
sgLAMC1#3
sgLAMC1#1
sgLAMC1#2
LAMC1
120 kDa
B-actin
40 kDa
h
EPD210FH
GFP % (dayN/day0)
sgControl
sgLAMC1#1
sgLAMC1#2
Day
i
EP1NS
GFP % (dayN/day0)
sgControl
sgLAMC1#1
sgLAMC1#2
Day
j
VBT372
GFP % (dayN/day0)
sgControl
sgLAMC1#1
sgLAMC1#2
Day
k
GBM2
GFP % (dayN/day0)
sgControl
sgLAMC1#1
sgLAMC1#2
Day

Supplementary Figure 6

a) Visualization of a translocation between chr1q and chr5p in the PFA ependymoma relapse sample RCEP2 R1.

b) Visualization of an inversion within chr1q that includes LAMC1 in the PFA ependymoma relapse tumor CHLAEP R1.

c) Barplot of RNA-seq expression analysis revealed that LAMC1 transcription is higher in the two 1q+ PFA ependymoma relapse (black color) than in primary PFA tumors with (pink) or without 1q gain (grey).

d) Barplots of *LAMC1* expression in tumor samples of the patients CHLAEP (left) and RCEP (right) demonstrating increased LAMC1 transcription in the relapse compared to the patient-matched primary tumors.

e) Barplot of transcription of LAMC1 across the INFORM cohort of 29 PFA ependymoma relapse tumors with (red) and without (white) 1q gain. The five samples with the highest LAMC1 expression were analyzed by WGS (black dashed box). Of those, the three samples with the highest LAMC1 expression have DNA breakpoints nearby the LAMC1 gene locus on chromosome 1q (+/-100kb, those samples are marked with stars).

f) Gene expression analysis of genes co-located with LAMC1 in the same neo-TAD comparing PFA relapse cases with (red) and without (white) 1q gain (INFORM cohort, n=29). Among these genes, only LAMC1 exhibits significant transcriptional upregulation in samples with 1q gain (p-value<0.05). The box plot center line, box limits, whiskers, and points indicate the median, upper/lower quartiles, 1.5× interquartile range and outliers, respectively.

g) Western blots show efficacy of sgRNAs targeting *LAMC1* and control sgRNA in EPD210FH cells. Mixture of different clones of infected EPD210FH cells are used to obtain protein extracts at day 5 post infection with indicated sgRNAs. B-actin is used as a loading control (n=1). *sgLAMC1#3 is excluded for further analysis.

h-k) Genetic (CRISPR-Cas9) time-course knockout of *LAMC1* in ependymoma PFA EPD210FH (h), ZFTA EP1NS (i) and VBT372 (j) as well as glioblastoma GBM2 cells (k) using a control sgRNA and two individual sgRNA constructs. All constructs are GFP tagged and GFP positive cells are sorted by FACS. Results are normalized to day 0 and data represent mean from n=2 independent experiments per cell line.

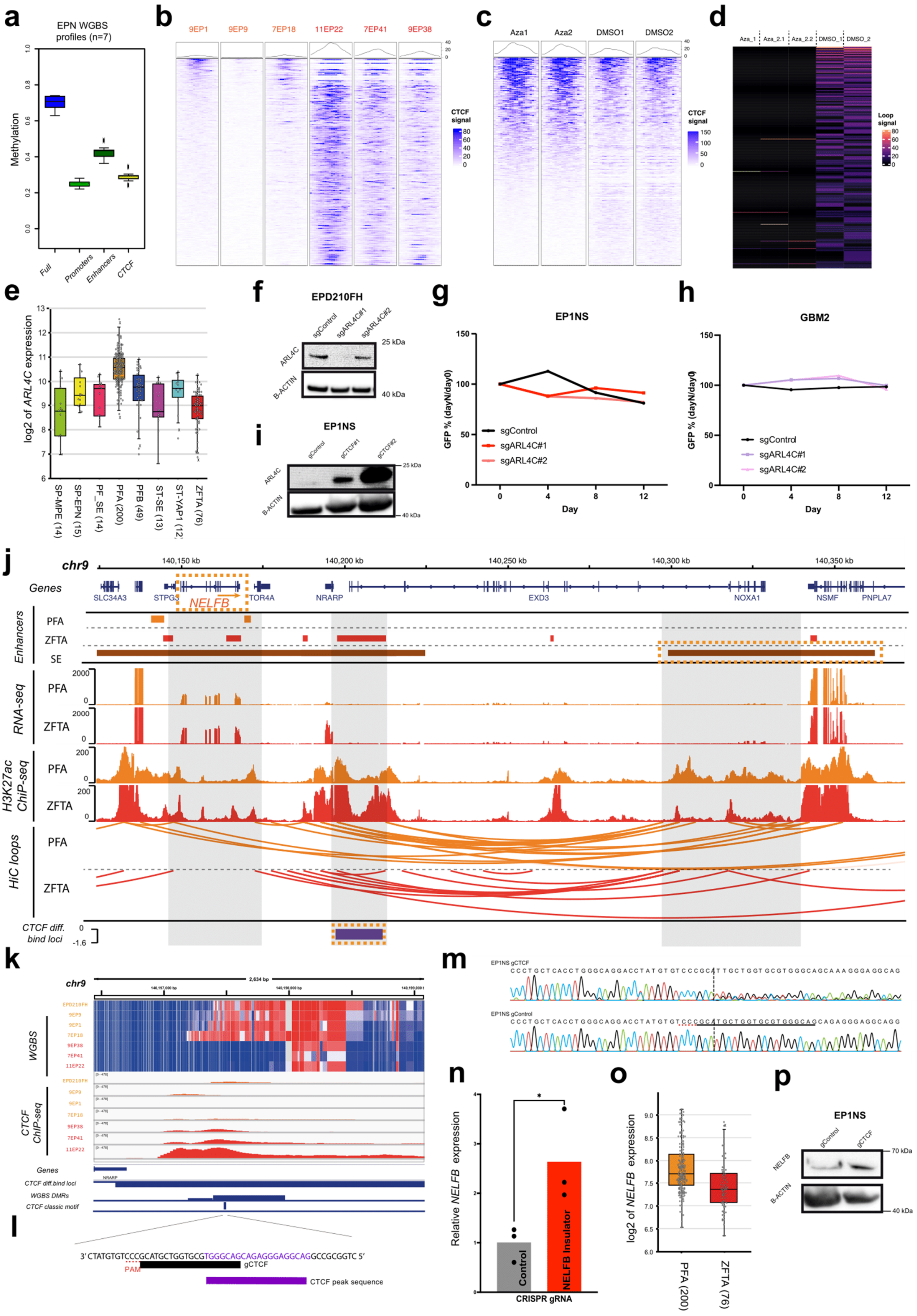
a
EPN WGBS profiles (n=7)
Methylation
Full
Promoters
Enhancers
CTCF
b
9EP1
9EP9
7EP18
11EP22
7EP41
9EP38
CTCF signal
c
Aza1
Aza2
DMSO1
DMSO2
CTCF signal
d
Aza_1
Aza_2.1
Aza_2.2
DMSO_1
DMSO_2
Loop signal
e
log2 of ARL4C expression
SP-MPE (14)
SP-EPN (15)
PF_SE (14)
PFA (200)
PFB (49)
ST-SE (13)
ST-YAP1 (12)
ZFTA (76)
f
EPD210FH
sgControl
sgARL4C#1
sgARL4C#2
ARL4C
B-ACTIN
25 kDa
40 kDa
g
EP1NS
GFP % (dayN/day0)
sgControl
sgARL4C#1
sgARL4C#2
Day
h
GBM2
GFP % (dayN/day0)
sgControl
sgARL4C#1
sgARL4C#2
Day
i
EP1NS
gControl
gCTCF#1
gCTCF#2
ARL4C
B-ACTIN
25 kDa
40 kDa
j
chr9
140,150 kb
140,200 kb
140,250 kb
140,300 kb
140,350 kb
Genes
SLC34A3
STPG3
NELFB
TOR4A
NRARP
EXD3
NOXA1
NSMF
PNPLA7
Enhancers
PFA
ZFTA
SE
RNA-seq
PFA
ZFTA
H3K27ac ChIP-seq
PFA
ZFTA
HiC loops
PFA
ZFTA
CTCF diff. bind loci
k
chr9
2,634 bp
WGBS
EPD210FH
9EP9
9EP1
7EP18
9EP38
7EP41
11EP22
CTCF ChIP-seq
Genes
NRARP
CTCF diff.bind loci
WGBS DMRs
CTCF classic motif
l
3' CTATGTGTCCCGCATGCTGGTGCGTGGGCAGCAGAGGGAGGCAGGCCGCGGTC 5'
PAM
gCTCF
CTCF peak sequence
m
EP1NS gCTCF
EP1NS gControl
n
Relative NELFB expression
Control
NELFB Insulator
CRISPR gRNA
o
log2 of NELFB expression
PFA (200)
ZFTA (76)
p
EP1NS
gControl
gCTCF
NELFB
B-ACTIN
70 kDa
40 kDa

Supplementary Figure 7

a) Mean methylation of PFA and RELA ependymoma tumors at promoters, enhancers and CTCF binding sites. The center line, box limits, whiskers and points indicate the median, upper/lower quartiles, 1.5× interquartile range and outliers, respectively.

b) Heatmap of CTCF ChIP-seq data at the 300 most significant differential CTCF binding sites comparing three PFA (left) and three RELA (right) ependymoma tumors.

c) Heatmap of CTCF Cut&Tag data at the 300 most significant differential CTCF binding sites comparing PFA cells treated with 5-Azacytidine and DMSO, respectively.

d) Heatmap of promoter capture Hi-C data at the 500 most significant differential DNA loops comparing PFA cells treated with 5-Azacytidine and DMSO, respectively. Loop signal is provided via size factor normalized read counts.

e) Boxplot showing *ARL4C* gene expression across ependymoma subgroups (Affymetrix gene expression data for n=393 ependymoma tumors). The center line, box limits, whiskers and points indicate the median, upper/lower quartiles, 1.5× interquartile range and outliers, respectively. *ARL4C* is significantly upregulated in PFA compared to the other ependymoma types (limma p-val.: 1.63e-16).

f) Western blots show efficacy of sgRNAs targeting *ARL4C* and control sgRNA in EPD210FH cells. Mixture of different clones of infected EPD210FH cells are used to obtain protein extracts at day 5 post infection with indicated sgRNAs. B-actin is used as a loading control (n=1).

g-h) Genetic (CRISPR-Cas9) time-course inhibition of *ARL4C* in ZFTA ependymoma (EP1NS) (g) and GBM (h) cells using a control sgRNA and two individual sgRNA constructs. All constructs are GFP tagged and GFP positive cells are sorted by FACS. Results are normalized to day 0 and data represent mean from n=2 independent experiments.

i) Representative Western blots show increased expression of ARL4C at the protein level after CRISPR-Cas9 targeting of CTCF binding sites using two different gRNAs, compared with control gRNA in ZFTA (EP1NS) cells. Mixture of different clones of infected EP1NS cells are used to obtain protein extracts after post infection with indicated sgRNAs. B-actin is used as a loading control (n=2).

j) Genome browser visualization of PFA ependymoma-specific DNA loops that associate ependymoma super enhancers with the NELFB gene locus.

k) WGBS-derived DNA methylation and CTCF ChIP-seq data from PFA and RELA ependymoma tumors show that a CTCF binding site separating the NELFB gene from the super-enhancer is replaced by DNA methylation in PFA tumors.

l) Detailed view of the CTCF motif targeted by CRISPR-Cas9: gRNA and protospacer adjacent motif (PAM) direct Cas9 nuclease to the motif.

m) Sequencing of the target site demonstrates the formation of indels (insertion or deletions).

n) qPCR reveals increased NELFB expression upon targeting the CTCF binding site in ZFTA ependymoma cells. Error bars represent mean ±SD (n=3). Error bars are derived from unpaired two tailed t-test calculated by GraphPad (p=0,0477).

o) Boxplot showing NELFB gene expression in PFA and ZFTA tumors (Affymetrix gene expression data for n=276 ependymoma tumors). The center line, box limits, whiskers and points indicate the median, upper/lower quartiles, 1.5× interquartile range and outliers, respectively. NELFB is significantly upregulated in PFA compared to ZFTA tumors (limma p-val.: 1.428e-05).

p) Western blots show increased expression of NELFB at the protein level by CRISPR-Cas9 either with gRNAs targeting CTCF motif or control sgRNA in EP1NS cells. Mixture of different clones of infected EP1NS cells are used to obtain protein extracts after post infection with indicated sgRNAs. B-actin is used as a loading control (n=1).
