# Supplementary material for: 3D genome mapping identifies subgroup-specific chromosome conformations and tumor-dependency genes in ependymoma
Source: Nat Commun. 2023 Apr 21;14:2300. doi: 10.1038/s41467-023-38044-0 (PMC10121654; doi:10.1038/s41467-023-38044-0)
Supplement: Supplementary file 3 — Description of Additional Supplementary Files [file 41467_2023_38044_MOESM3_ESM.pdf]

Description of Additional Supplementary Files

File Name: Supplementary Data 1
Description: Cohort of ependymoma tumor samples and available data types.

File Name: Supplementary Data 2
Description: Enhancer associated genes supported by DNA loops in (a) PFA and (b) ZFTA ependymomas.

File Name: Supplementary Data 3
Description: Structural variants in ZFTA (a,b) and PFA (c,d) ependymoma samples as identified by (a,c) hicBreakFinder and Hi-C SV/trans (b,d) tools.

File Name: Supplementary Data 4
Description: DAVID Gene Ontology analysis results for differentially expressed genes specific for PFA compared to other ependymoma subgroups.

File Name: Supplementary Data 5
Description: (a) Enhancer-associated genes supported by DNA loops that are potentially formed due to the replacement of CTCF binding sites by DNA methylation in PFA ependymoma tumors. (b) Genes in connection ependymoma superenhancers by DNA loops that are potentially formed due to hypermethylation and loss of CTCF binding sites in PFA ependymoma tumors.

File Name: Supplementary Data 6
Description: Overview of shRNA, sgRNA and primer sequences applied in experimental validation.

File Name: Supplementary Data 7
Description: Drugs selected for experimental validation

File Name: Supplementary Data 8
Description: Overview of antibodies applied in experimental validation.

File Name: Supplementary Data 9
Description: Components of cell culture media.
