# Supplementary material for: 3D genome mapping identifies subgroup-specific chromosome conformations and tumor-dependency genes in ependymoma
Source: Nat Commun. 2023 Apr 21;14:2300. doi: 10.1038/s41467-023-38044-0 (PMC10121654; doi:10.1038/s41467-023-38044-0)
Supplement: Supplementary file 5 — Reporting Summary [file 41467_2023_38044_MOESM5_ESM.pdf]

nature portfolio

Corresponding author(s): Lukas Chavez

Last updated by author(s): Mar 1, 2023

Reporting Summary

Nature Portfolio wishes to improve the reproducibility of the work that we publish. This form provides structure for consistency and transparency in reporting. For further information on Nature Portfolio policies, see our Editorial Policies and the Editorial Policy Checklist.

Statistics

For all statistical analyses, confirm that the following items are present in the figure legend, table legend, main text, or Methods section.

n/a	Confirmed	
☐	☒	The exact sample size (*n*) for each experimental group/condition, given as a discrete number and unit of measurement
☐	☒	A statement on whether measurements were taken from distinct samples or whether the same sample was measured repeatedly
☐	☒	The statistical test(s) used AND whether they are one- or two-sided<br>*Only common tests should be described solely by name; describe more complex techniques in the Methods section.*
☐	☒	A description of all covariates tested
☐	☒	A description of any assumptions or corrections, such as tests of normality and adjustment for multiple comparisons
☐	☒	A full description of the statistical parameters including central tendency (e.g. means) or other basic estimates (e.g. regression coefficient) AND variation (e.g. standard deviation) or associated estimates of uncertainty (e.g. confidence intervals)
☒	☐	For null hypothesis testing, the test statistic (e.g. *F*, *t*, *r*) with confidence intervals, effect sizes, degrees of freedom and *P* value noted<br>*Give P values as exact values whenever suitable.*
☒	☐	For Bayesian analysis, information on the choice of priors and Markov chain Monte Carlo settings
☐	☒	For hierarchical and complex designs, identification of the appropriate level for tests and full reporting of outcomes
☐	☒	Estimates of effect sizes (e.g. Cohen's *d*, Pearson's *r*), indicating how they were calculated

Our web collection on statistics for biologists contains articles on many of the points above.

Software and code

Policy information about availability of computer code

Data collection

Affymetrix gene expression data was collected from the dataset "Tumor Ependymoma (DKFZ - Public) - Kool - 626 - MAS5.0 - u133p2" from R2 platform (http://r2.amc.nl)

Data analysis

Initial HiC sequencing data processing was performed using HiCPro 2.9.0 toolkit.
Main visualization and normalized full contacts extraction was performed with JuiceBox v0.7.5 toolkit . Normalization for clustering was performed with Genome Contact Map Explorer v1.0.9.
Per sample loop calling was applied from FitHiC v2.0.6 method on bin sizes 5 Kbp with maximum distance between bins 50 Mbp.
TAD calling was performed based on 50 Kbp bins resolution using TopDom v1 tool.
Loops analysis was performed with packages: InTAD 1.9.2, diffloop 1.10
SV discovery from HiC data was done with hicBreakFinder v1 tool.
SV calling on WGS data was performed with Delly 0.6.7. Fusion calling was performed on RNA-seq data with tool InFusion v0.6.2.
WGBS data initial processing was performed with methylCtools v0.9.4, while DMR calling between PFA and ZFTA groups was done with metilene v0.2.6 tool. CTCF ChIP-seq reads were aligned to hg19 reference with BWA v0.5.10 and peak calling was performed using Macs v1.463. Differential RELA peaks between EPN PFA and RELA were detected using DiffBind 3.7 R package. Visualization of WGBS signals in diff peaks loci was performed with ComplexHeatmap R package v2.12.
All data analysis steps/cluster submission commands were generated from custom R and Python scripts that are collected public github repository: https://github.com/kokonech/EPN_HiC_analysis

For manuscripts utilizing custom algorithms or software that are central to the research but not yet described in published literature, software must be made available to editors and reviewers. We strongly encourage code deposition in a community repository (e.g. GitHub). See the Nature Portfolio guidelines for submitting code & software for further information.

Data

Policy information about availability of data

All manuscripts must include a data availability statement. This statement should provide the following information, where applicable:

- Accession codes, unique identifiers, or web links for publicly available datasets
- A description of any restrictions on data availability
- For clinical datasets or third party data, please ensure that the statement adheres to our policy

The novel sequencing data raw materials (Hi-C, CTCF, WGBS) generated in this study have been deposited in the European Genome-phenome archive (https://www.ebi.ac.uk/ega/home) under the accession code: EGAS00001002696; this source already contains other data types (RNA-seq, H3K27ac) for the corresponding target tumor samples. The Affymetrix data used in this study are available in the GEO database under accession codes GSE64415, GSE50161, GSE50385, GSE21687, GSE3526.

Human research participants

Policy information about studies involving human research participants and Sex and Gender in Research.

Reporting on sex and gender	Sex or age of the cancer patients is not considered in study design.
Population characteristics	19 patients with diagnosis ependymoma tumors
Recruitment	The tumor materials were collected from international cohorts
Ethics oversight	Ethics of materials availability is verified in previous studies, the description is provided in the corresponding study: https://www.nature.com/articles/nature25169

Note that full information on the approval of the study protocol must also be provided in the manuscript.

Field-specific reporting

Please select the one below that is the best fit for your research. If you are not sure, read the appropriate sections before making your selection.

☒ Life sciences ☐ Behavioural & social sciences ☐ Ecological, evolutionary & environmental sciences

For a reference copy of the document with all sections, see nature.com/documents/nr-reporting-summary-flat.pdf

Life sciences study design

All studies must disclose on these points even when the disclosure is negative.

Sample size	Main biological comparisons were performed among materials either from tumor samples or cell lines, representing distinct ependymoma sugbroups. Each target subgroup had at least 2 samples/repeats in all comparisons for verification.
Data exclusions	No data were excluded from the analysis.
Replication	All sequencing datasets (HiC, CTCF, WGBS) for comparison among cohorts had at least n=3 samples per group. All attempts at replication were successful and described in the manuscript.
Randomization	Due to limits in ependymoma tumors main focus was comparison between the tumor subgroups. Randomization in the study was achieved from selection of additional negative control for data analysis (e.g. HiC and gene expression data from normal brain tissues) and experimental validation (e.g. glioblastoma tumor cell lines)
Blinding	Blinding was not relevant in our study since experimental validation was focused on specific tumor cell lines with limited variance e.g. only 2 cell lines avaiilable per group

Reporting for specific materials, systems and methods

We require information from authors about some types of materials, experimental systems and methods used in many studies. Here, indicate whether each material, system or method listed is relevant to your study. If you are not sure if a list item applies to your research, read the appropriate section before selecting a response.

Materials & experimental systems

n/a	Involved in the study
☐	☒ Antibodies
☐	☒ Eukaryotic cell lines
☒	☐ Palaeontology and archaeology
☒	☐ Animals and other organisms
☒	☐ Clinical data
☒	☐ Dual use research of concern

Methods

n/a	Involved in the study
☐	☒ ChIP-seq
☐	☒ Flow cytometry
☒	☐ MRI-based neuroimaging

Antibodies

Antibodies used

The information about the antibodies used in this study is : Beta-actin HRP (Abcam, Cat# ab49900, 1:10000) RCOR2 (ptGlab, Cat# 239691-AP, 1:1000) LSD1 (Abcam, Cat# ab129195, 1:10000, Lot#GR244503-20) LAMC1 (Sigma, Cat# Sigma HPA001909, 1:1000, Lot#A114781) ARL4C (Sigma, Cat#HPA028927, 1:1000 for western, 1:200 for IF, Lot#C115998), MAP3K20 (Bethyl, Cat#A301-993A,1:1000 Lot#1), NELFB (Bethyl, Cat#A301-912A, 1:2000, Lot#1), Anti-rabbit Ig HRP linked (Cell Signaling, Cat#7074S, 1:2500, Lot#32), CTCF (Active Motif, Cat#39357, 5 ug per ChIP), AlexaFluor 568 (Thermo Fisher, Cat#A10042, 1:400)

Validation

The only antibody was validated by our collaborators and the manufacturer is below:
RCOR2 : https://pubmed.ncbi.nlm.nih.gov/32284543/
The following antibodies were previously tested in several published manuscripts.
Beta-actin, LSD1, Anti-rabbit Ig HRP linked, CTCF, AlexaFluor 568
The following antibodies were quality checked in the manufacture's website:
LAMC1: https://www.sigmaaldrich.com/catalog/product/sigma/hpa001909?lang=de&region=DE
ARL4C : https://www.sigmaaldrich.com/catalog/product/sigma/hpa028927?lang=de&region=DE
MAP3K20 : https://www.fortislife.com/products/primary-antibodies/rabbit-anti-zak-antibody/BETHYL-A301-993
NELFB : https://www.fortislife.com/products/primary-antibodies/rabbit-anti-cobra1-antibody/BETHYL-A301-912

Eukaryotic cell lines

Policy information about cell lines and Sex and Gender in Research

Cell line source(s)

HEK293T from Lena Kutscher Lab, DKFZ . SU-pcGBM2 histone wt (called briefly GBM2 in this study) from David T. Jones lab, DKFZ. EP1NS and BT-165 patient-derived cell lines were established in Heidelberg. EPD210FH from James Olson lab, Seattle. BT214 and VBT372 patient-derived cell lines were provided by Johannes Gojo lab, Vienna. ST-1, PFA2-4-9 patient-derived cell lines were established in Micheal Taylor lab, Toronto. Human astrocytes was bought from Gibco (N7805100).

Authentication

All patient-derived primary ependymoma culture were checked for DNA methylation and compared with primary tumor taken from patient. GBM2 glioblastoma and HEK293T cell lines were not authenticated.

Mycoplasma contamination

All cell cultures were routinely tested and confirmed to be free of Mycoplasma contamination using a Mycoplasma detection PCR strategy with positive and negative controls.

Commonly misidentified lines
(See ICLAC register)

No commonly misidentified cell lines were used in the study.

ChIP-seq

Data deposition

☒ Confirm that both raw and final processed data have been deposited in a public database such as GEO.

☒ Confirm that you have deposited or provided access to graph files (e.g. BED files) for the called peaks.

Data access links
May remain private before publication.

Genome-phenome archive (https://www.ebi.ac.uk/ega/home) under the accession number: GAS00001002696

Files in database submission

Full list of files can be found under the accession number provided above.

Genome browser session
(e.g. UCSC)

IGV online sessions are combined of HiC loops, CTCF ChIP-seq peaks, H372Kac ChIP-seq peaks and RNA-seq data tracks and available at public github repository: https://github.com/kokonech/EPN_HiC_analysis/wiki

Methodology

Replicates

Ependymoma tumor subgroups were represented by the following number of samples: PFA - 4, RELA - 3

Sequencing depth

The sequencing procedure for tumor samples was performed with Illumina HiSeq 2000 (read size 125 bp) and resulted in 722722954

Sequencing depth	paired-end reads in total (~103246136 mean reads per sample). From them, ~90% reads were mapped with formation of correct pairs to hg19 reference. Further, in order to prepare for the peaks calling procedure, duplicates removal was performed for each sample alignment using samtools v0.1.18.
Antibodies	CTCF antibody
Peak calling parameters	Peaks computation was performed with MACS v1.4 tool using whole genome sequencing data from the same samples as background control and filtered with min p-value limit 1e-9.
Data quality	The quality control was performed with Qualimap v2.2.1 tool mode BAM QC applied for each alignment file to measure mapped reads proportion, coverage and insert size.
Software	Differential peak calling was performed using R package DiffBind 2.6.6

Flow Cytometry

Plots

Confirm that:

☒ The axis labels state the marker and fluorochrome used (e.g. CD4-FITC).

☒ The axis scales are clearly visible. Include numbers along axes only for bottom left plot of group (a 'group' is an analysis of identical markers).

☒ All plots are contour plots with outliers or pseudocolor plots.

☒ A numerical value for number of cells or percentage (with statistics) is provided.

Methodology

Sample preparation	For competitive growth assay, cells were treated with Accutase, spindown the pellet and resuspended in corrresponding fresh media (100-150 ul). For Annexin V apoptosis assay, cells were treated with Accutase, washed two times with assay buffer and stained according to instruction from company without any modifications.
Instrument	BD FACS Fortessa (DKFZ Facs facility, TP3) : Annexin V apoptosis assay, competetive growth assay for GFP+ or RFP+ cell populations.<br>BD Facs Canto (DKFZ Facs facility, TP3) : Competitive growth assay for GFP+ populations
Software	FACS data was analyzed with Flow Jo version 10.7 and 10.8
Cell population abundance	For competitive growth assay, viable cell population was determined between FSC-A and SSC-A axis. Next, single cell population was gated between FSC-H and FSC-A axis. Then, minumum 5000 events were recorded for all samples. Afterwords, GFP+ or RFP+ cell population was determined.<br>For Annexin V apoptosis assay, viable cells determination was skipped, started from single cells gated population. Next, GFP+ cell population was determined and finally apoptotic fraction was calculated. Minumum 10000 events were recorded for GFP + population for this analysis.
Gating strategy	For competitive growth assay, there is no labeling for marker identification. Therefore, gating was determined based on florescence positive (GFP or RFP) and negative cell population.<br>For Annexin V apoptosis assay, no dead cells were excluded in single cell gating. However, apoptotic fraction was determined within GFP+ population for each sample by looking at Annexin V + PI- cell population.

☒ Tick this box to confirm that a figure exemplifying the gating strategy is provided in the Supplementary Information.
