# Supplementary material for: 3D genome mapping identifies subgroup-specific chromosome conformations and tumor-dependency genes in ependymoma
Source: Nat Commun. 2023 Apr 21;14:2300. doi: 10.1038/s41467-023-38044-0 (PMC10121654; doi:10.1038/s41467-023-38044-0)
Supplement: Supplementary file 6 — Source Data [file 41467_2023_38044_MOESM6_ESM.zip › Source Data Western blots 270223.pdf]

Uncropped and unprocessed blots

Supplementary figure 3d

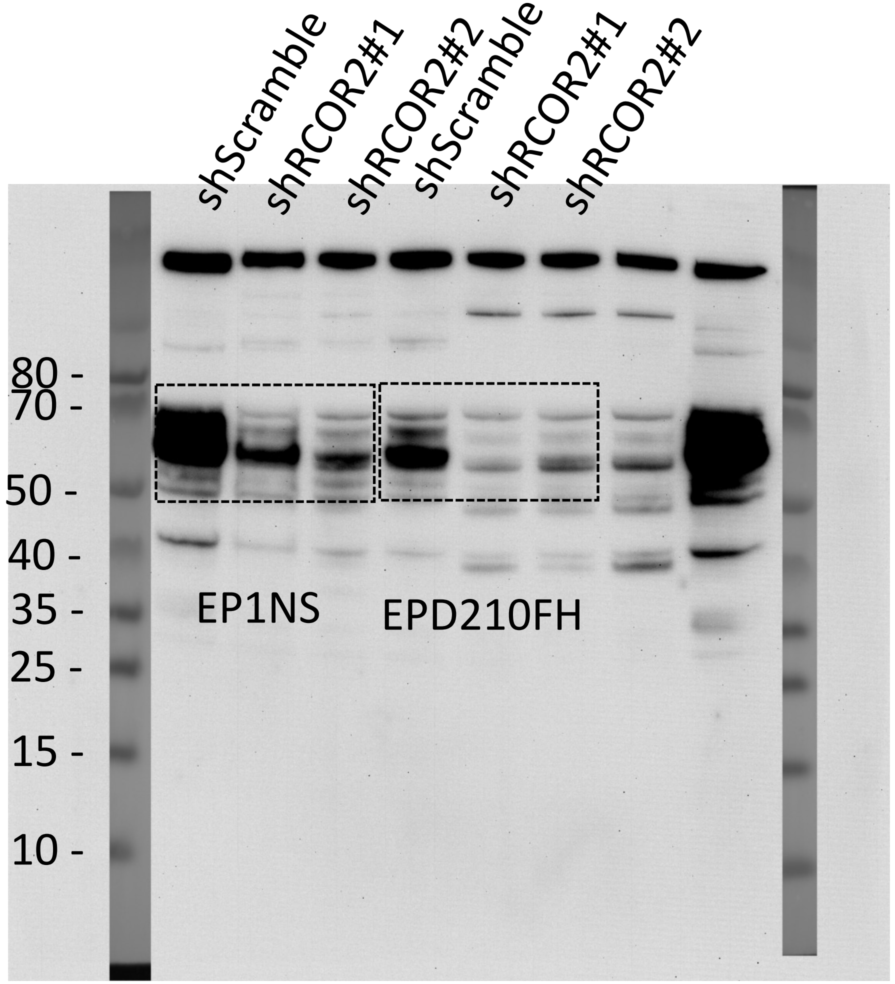

RCOR2

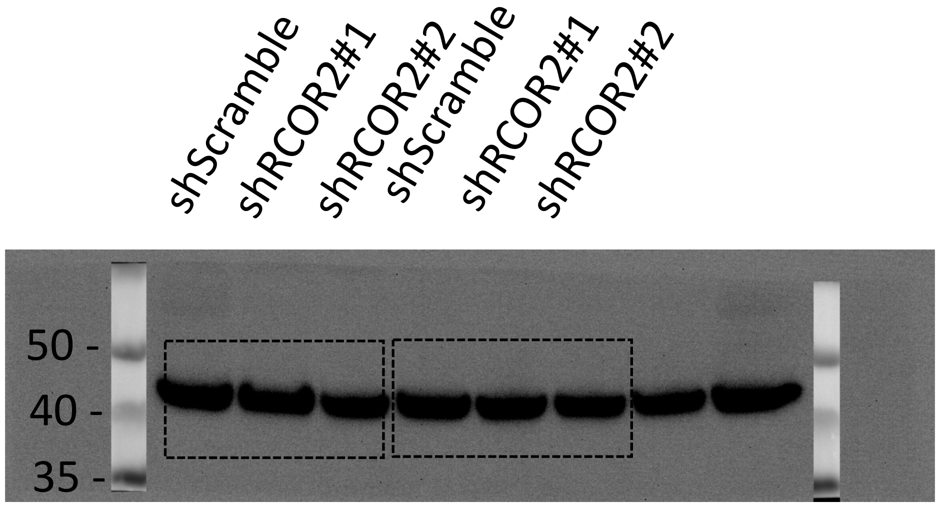

B-actin after cutting and stripping the same membrane

Supplementary figure 3d

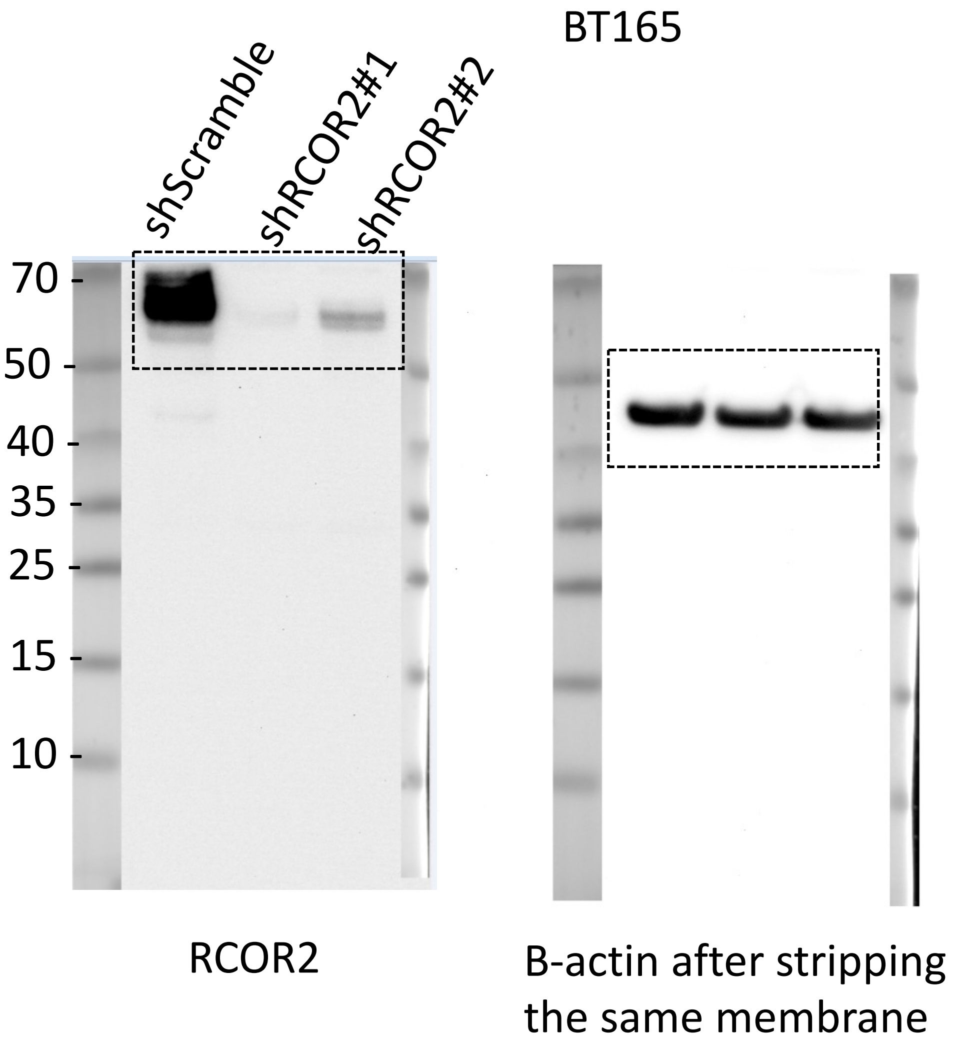

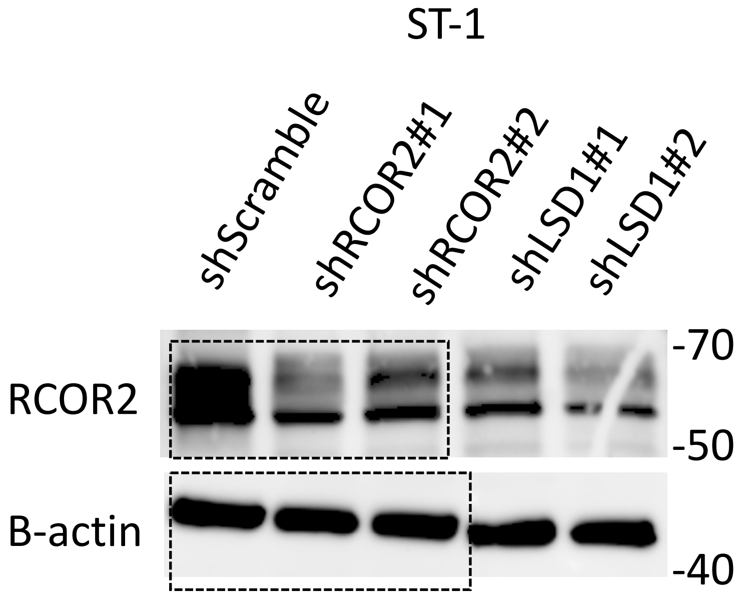

Supplementary figure 3e

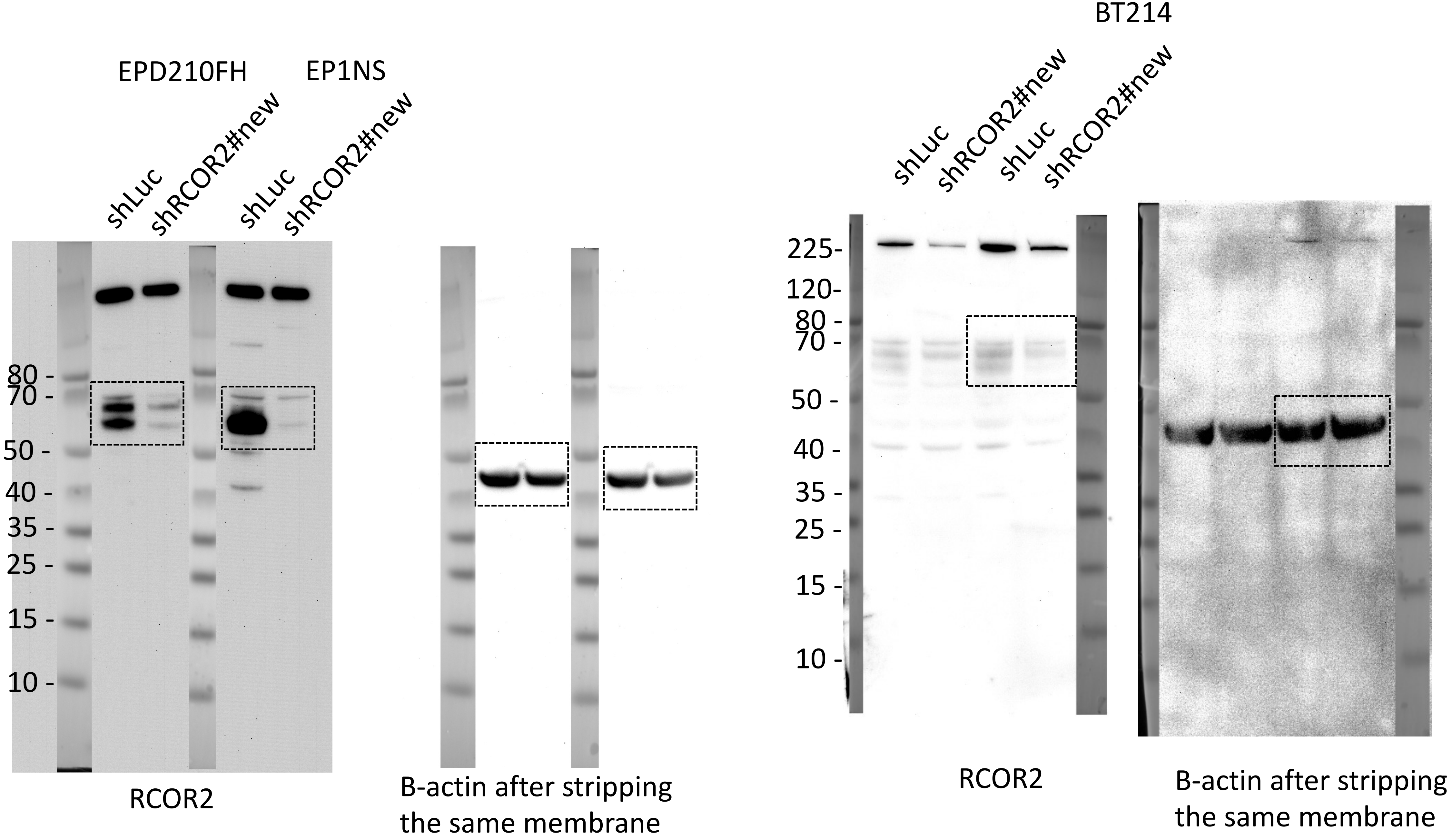

Supplementary figure 3i

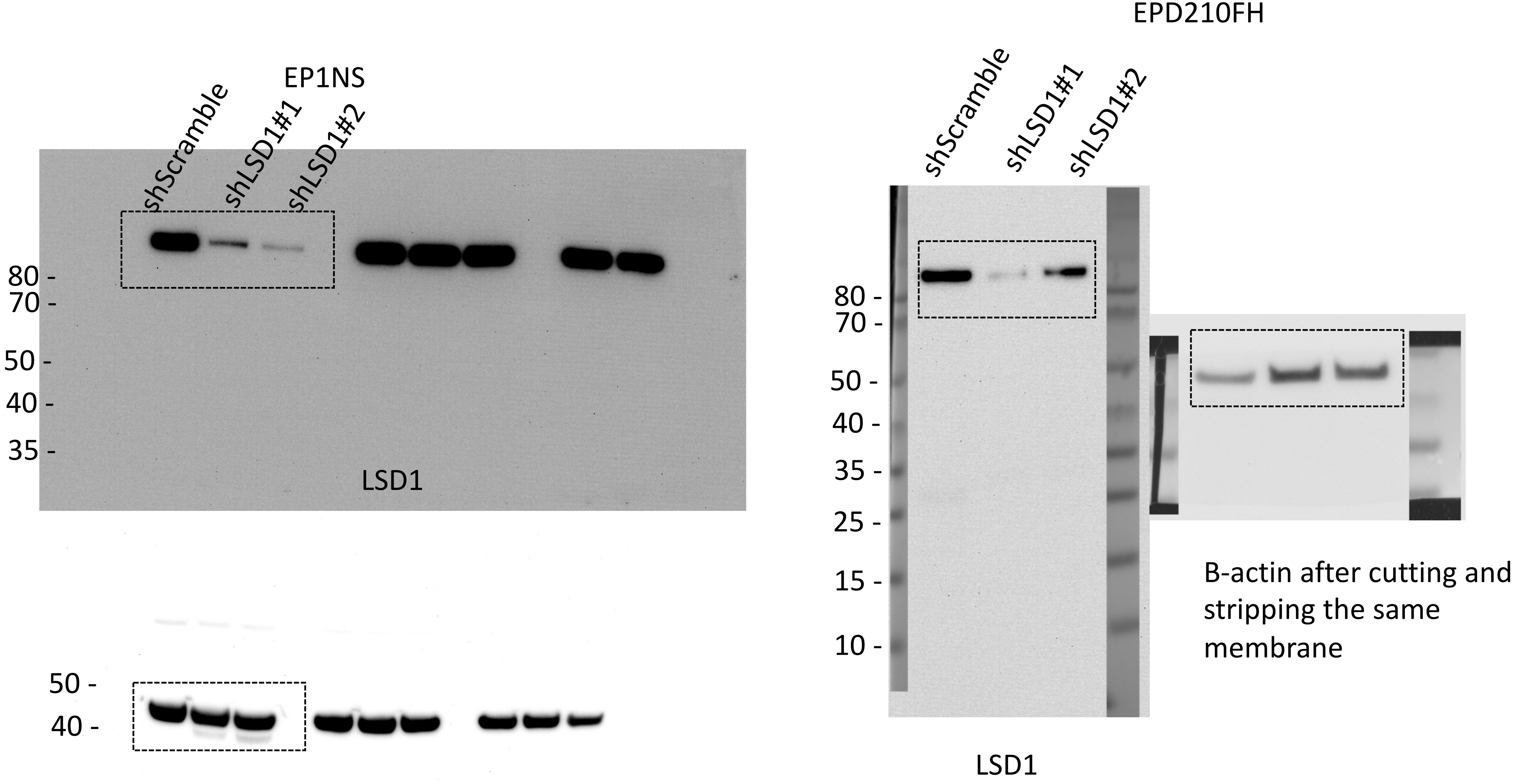

B-actin after stripping the same membrane

Supplementary figure 3i

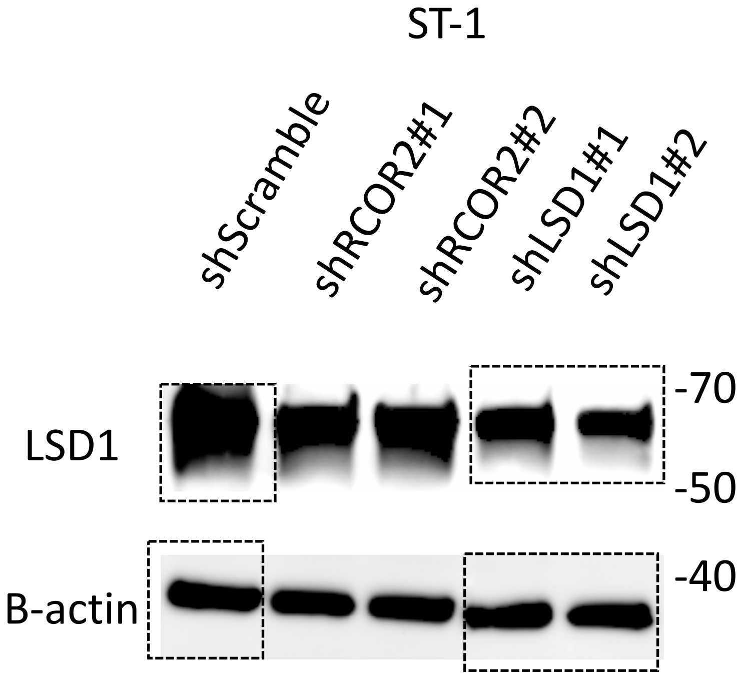

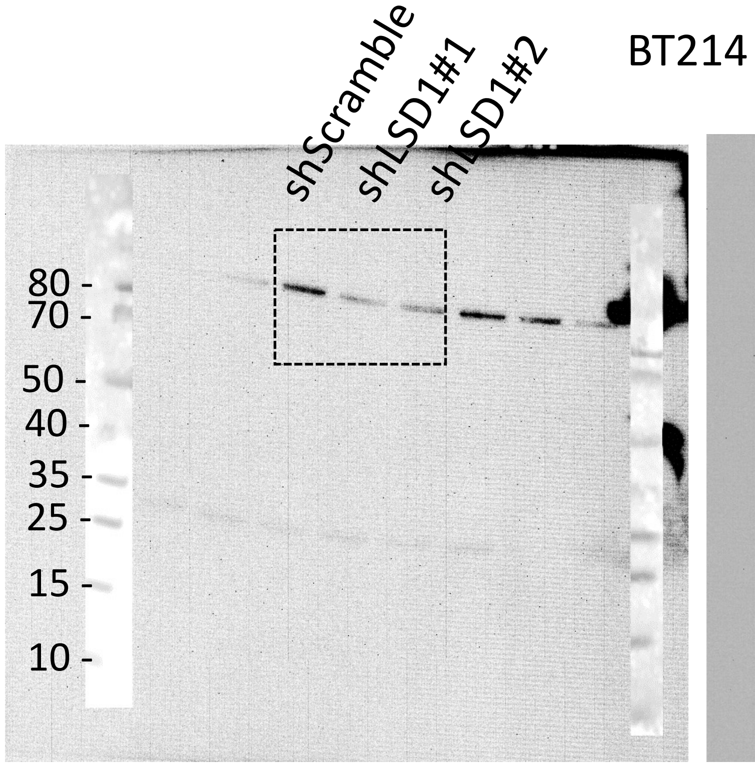

LSD1

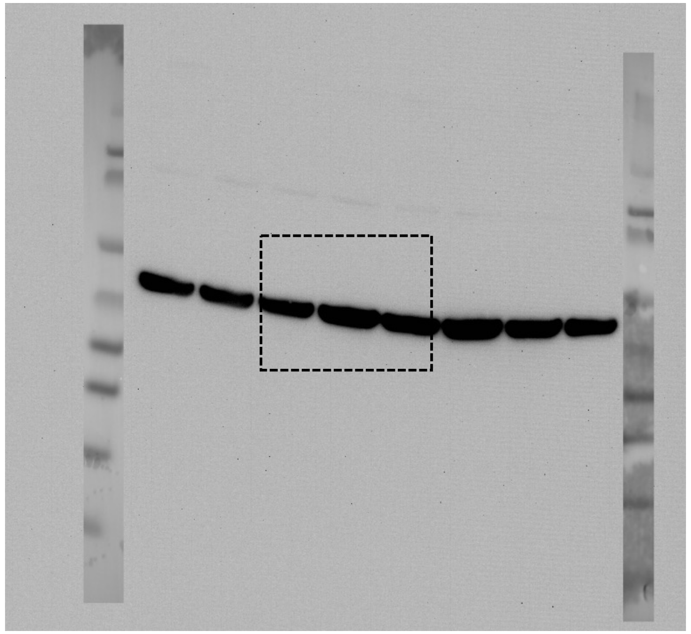

B-actin after stripping the same membrane

Supplementary figure 4d

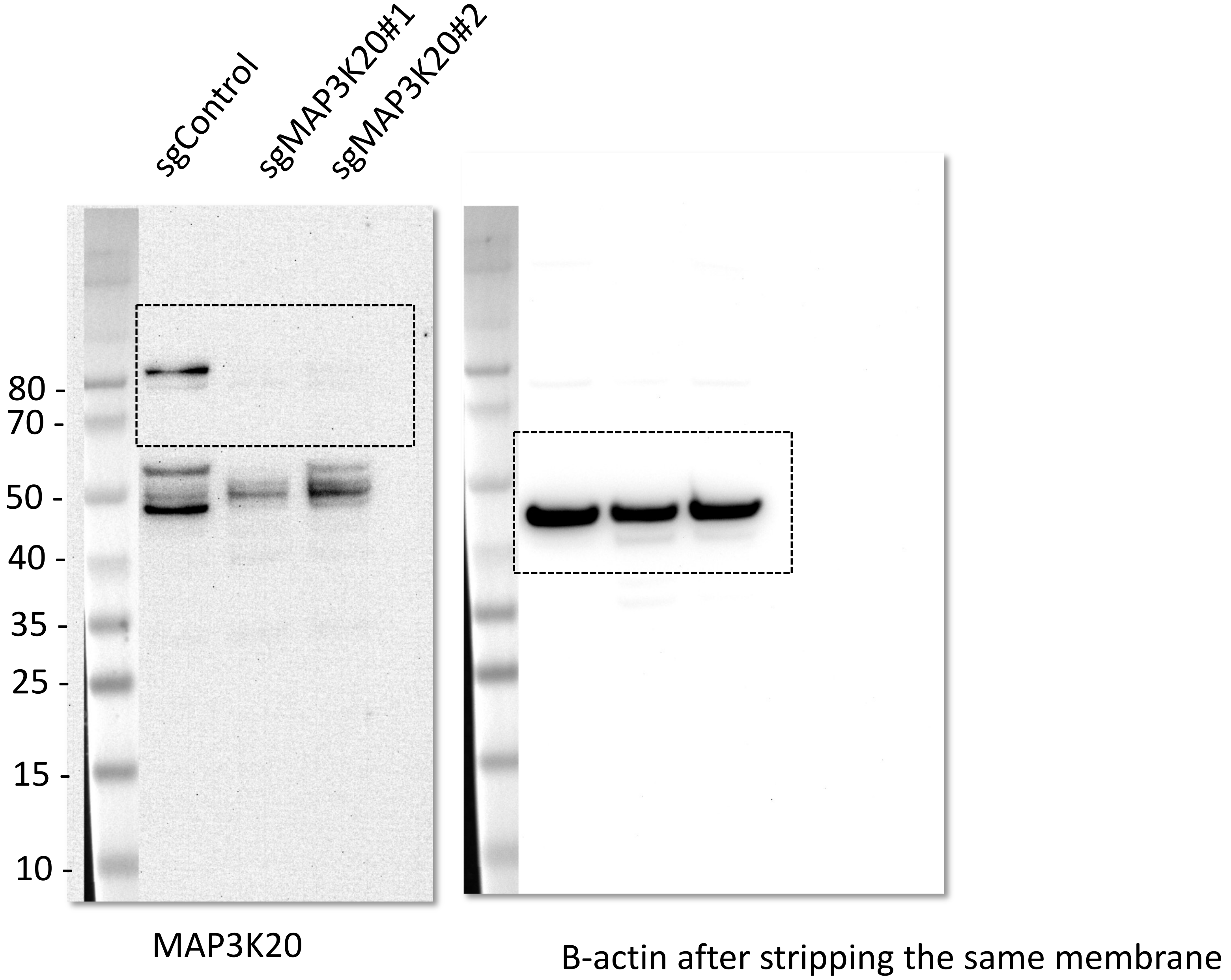

Supplementary figure 6g

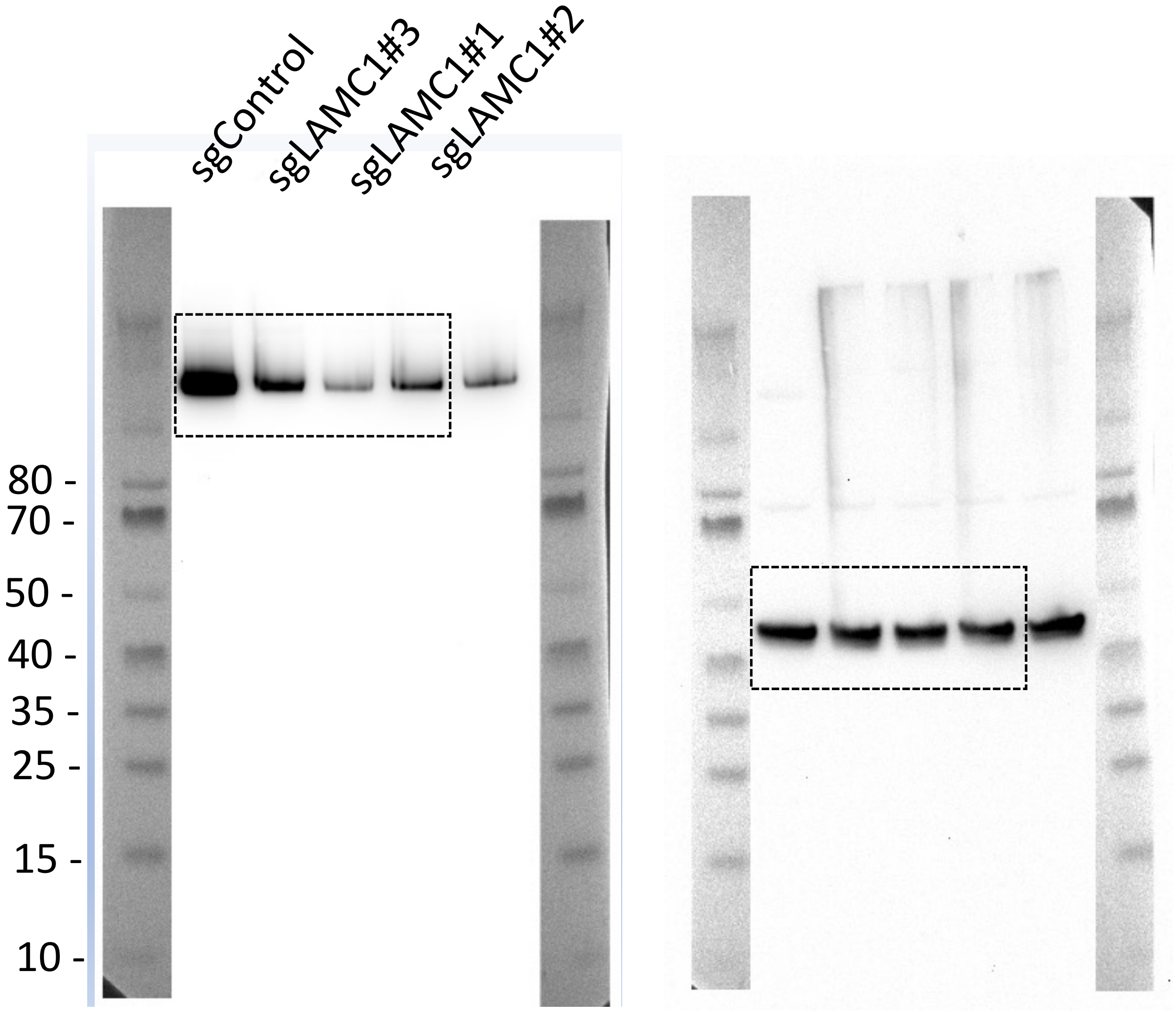

LAMC1

B-actin after stripping the same membrane

Supplementary figure 7f

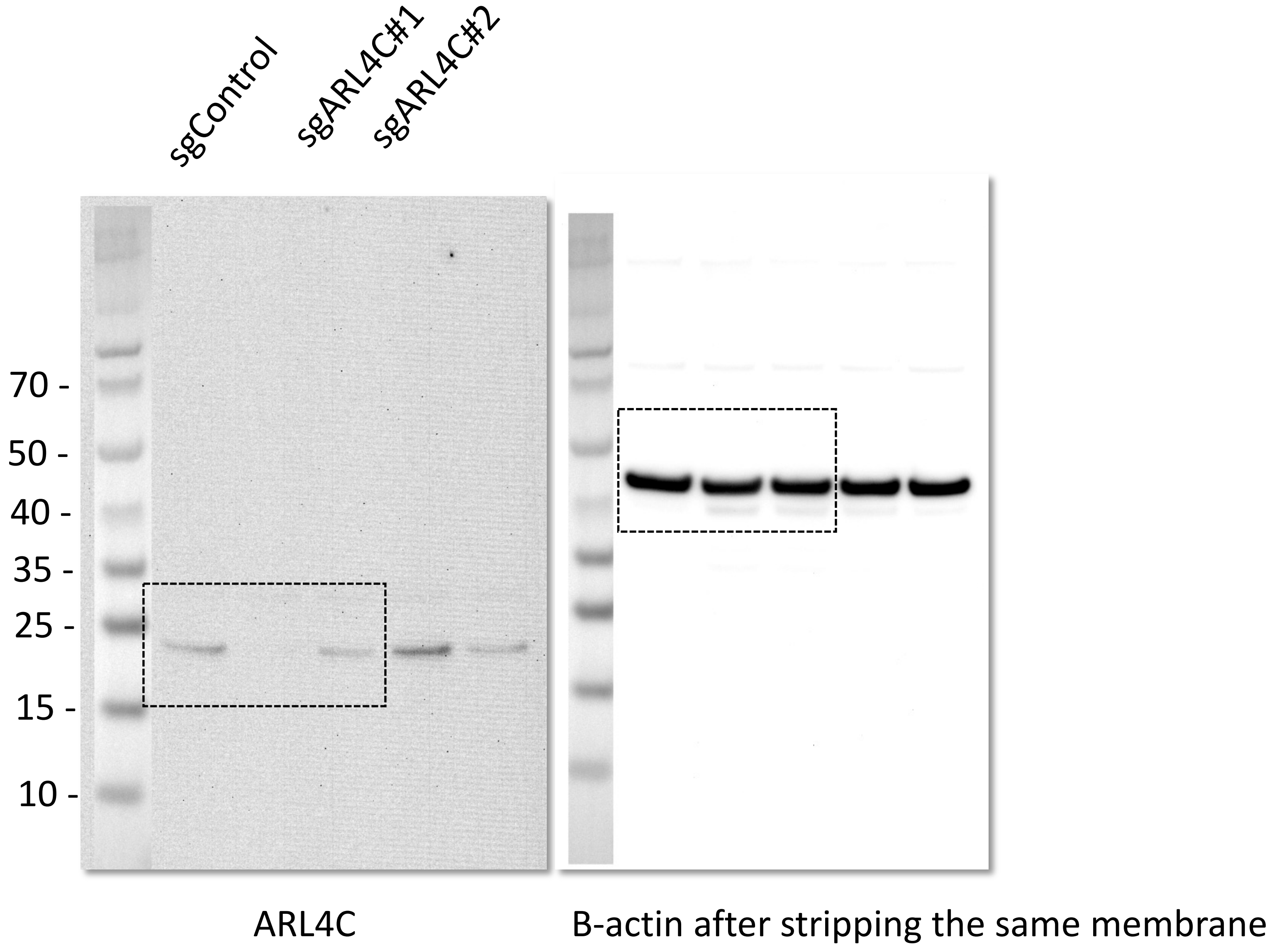

Supplementary figure 7i

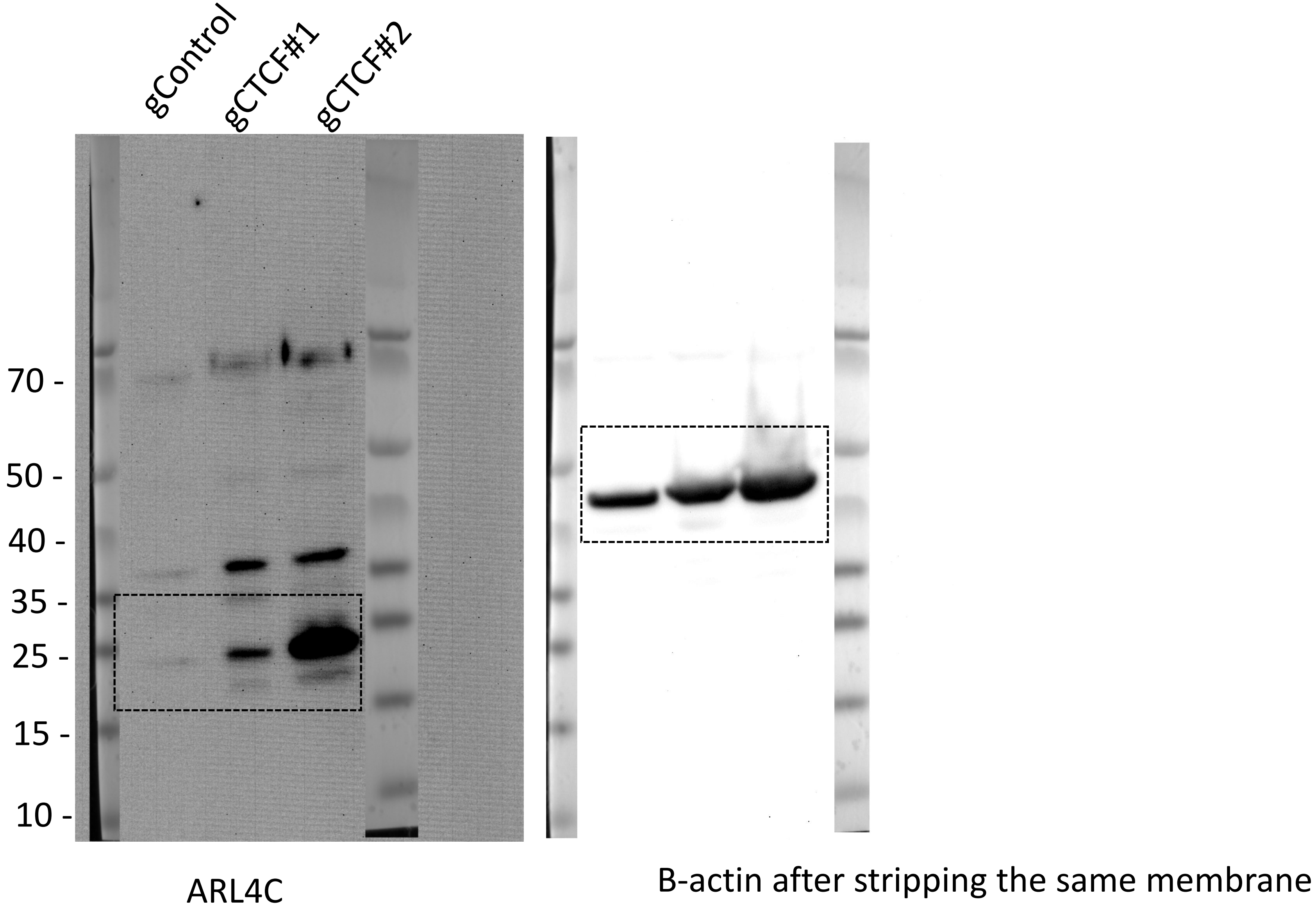

Supplementary figure 7p

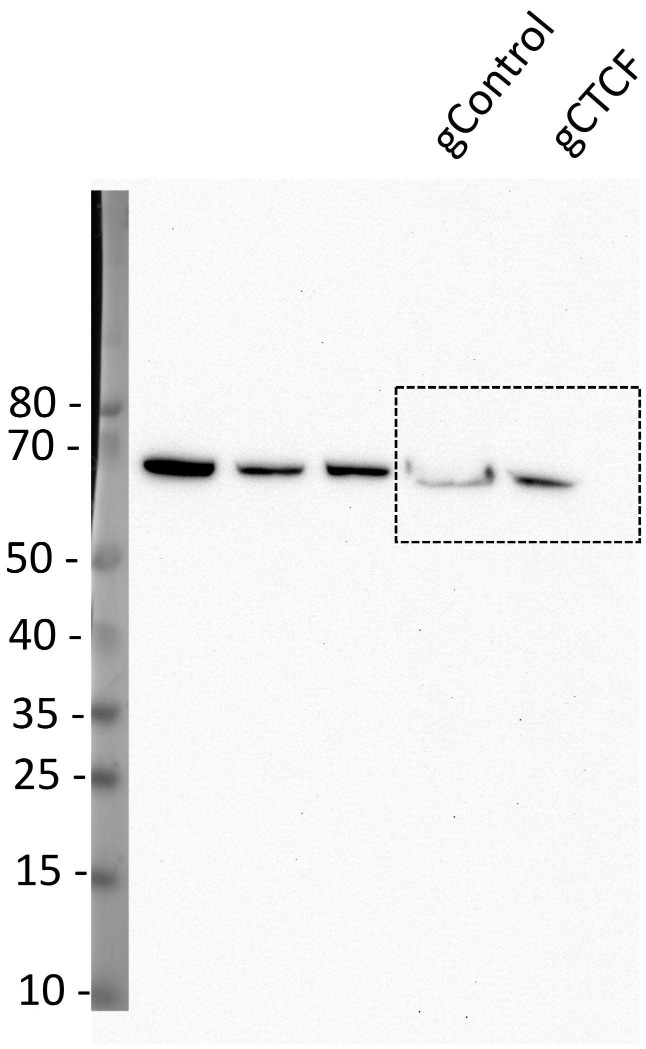

NELFB

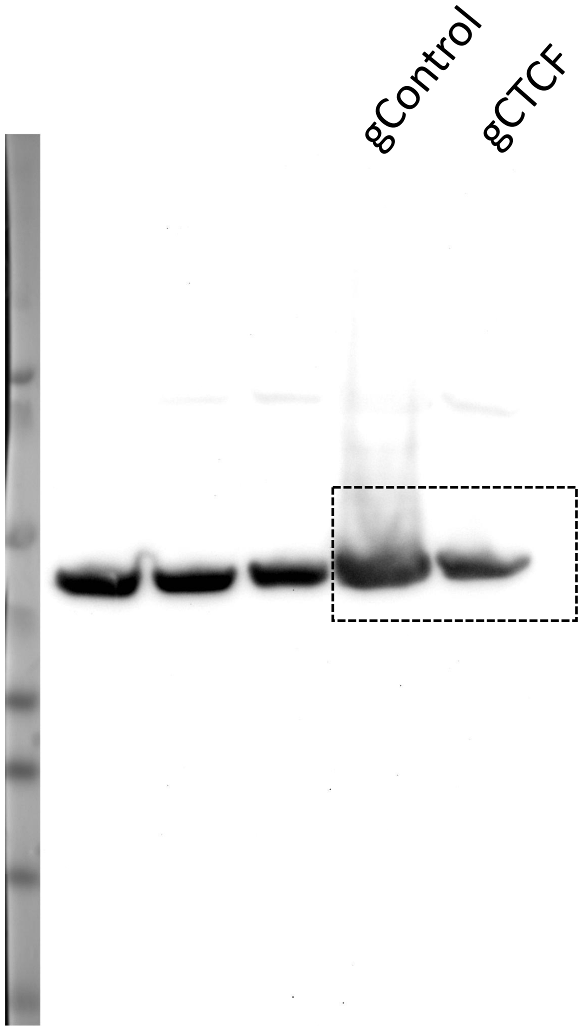

B-actin after stripping the same membrane
